# Myocardin reverses insulin resistance and ameliorates cardiomyopathy by increasing IRS-1 expression in a murine model of lipodystrophy caused by adipose deficiency of vacuolar H^+^-ATPase V0d1 subunit

**DOI:** 10.7150/thno.93192

**Published:** 2024-03-11

**Authors:** Wenlin Yuan, Hui Lin, Yuan Sun, Lihuan Liu, Meijuan Yan, Yujuan Song, Xiaofan Zhang, Xiangling Lu, Yipei Xu, Qiyue He, Kunfu Ouyang, Chenglin Zhang, Yong Pan, Yu Huang, Ying Li, Xifeng Lu, Jie Liu

**Affiliations:** 1 Department of pathophysiology, Shenzhen University Medical School, Shenzhen University, Shenzhen, China.; 2 Clinical Research Center, The First Affiliated Hospital of Shantou University Medical College, Shantou, China.; 3 Division of Pharmacology and Vascular Medicine, Department of Internal Medicine, Erasmus Medical Center, Rotterdam, the Netherlands.; 4 Department of Pharmacology, Shantou University Medical College, Shantou, China.; 5 Department of Pharmacology, College of Pharmacy, Shenzhen Technology University, Shenzhen, China.; 6 Department of Cardiovascular Surgery, Peking University Shenzhen Hospital, Shenzhen, China.; 7 Department of Biomedical Sciences, City University of Hong Kong, Hong Kong, China.

## Abstract

**Aim:** Adipose tissue (AT) dysfunction that occurs in both obesity and lipodystrophy is associated with the development of cardiomyopathy. However, it is unclear how dysfunctional AT induces cardiomyopathy due to limited animal models available. We have identified vacuolar H^+^-ATPase subunit V_o_d1, encoded by *Atp6v0d1*, as a master regulator of adipogenesis, and adipose-specific deletion of *Atp6v0d1* (*Atp6v0d1*^AKO^) in mice caused generalized lipodystrophy and spontaneous cardiomyopathy. Using this unique animal model, we explore the mechanism(s) underlying lipodystrophy-related cardiomyopathy.

**Methods and Results:** *Atp6v0d1*^AKO^ mice developed cardiac hypertrophy at 12 weeks, and progressed to heart failure at 28 weeks. The *Atp6v0d1*^AKO^ mouse hearts exhibited excessive lipid accumulation and altered lipid and glucose metabolism, which are typical for obesity- and diabetes-related cardiomyopathy. The *Atp6v0d1*^AKO^ mice developed cardiac insulin resistance evidenced by decreased IRS-1/2 expression in hearts. Meanwhile, the expression of forkhead box O1 (FoxO1), a transcription factor which plays critical roles in regulating cardiac lipid and glucose metabolism, was increased. RNA-seq data and molecular biological assays demonstrated reduced expression of myocardin, a transcription coactivator, in *Atp6v0d1*^AKO^ mouse hearts. RNA interference (RNAi), luciferase reporter and ChIP-qPCR assays revealed the critical role of myocardin in regulating IRS-1 transcription through the CArG-like element in IRS-1 promoter. Reducing IRS-1 expression with RNAi increased FoxO1 expression, while increasing IRS-1 expression reversed myocardin downregulation-induced FoxO1 upregulation in cardiomyocytes. *In vivo*, restoring myocardin expression specifically in *Atp6v0d1*^AKO^ cardiomyocytes increased IRS-1, but decreased FoxO1 expression. As a result, the abnormal expressions of metabolic genes in *Atp6v0d1*^AKO^ hearts were reversed, and cardiac dysfunctions were ameliorated. Myocardin expression was also reduced in high fat diet-induced diabetic cardiomyopathy and palmitic acid-treated cardiomyocytes. Moreover, increasing systemic insulin resistance with rosiglitazone restored cardiac myocardin expression and improved cardiac functions in *Atp6v0d1*^AKO^ mice.

**Conclusion:** *Atp6v0d1*^AKO^ mice are a novel animal model for studying lipodystrophy- or metabolic dysfunction-related cardiomyopathy. Moreover, myocardin serves as a key regulator of cardiac insulin sensitivity and metabolic homeostasis, highlighting myocardin as a potential therapeutic target for treating lipodystrophy- and diabetes-related cardiomyopathy.

## Introduction

Cardiovascular diseases (CVDs), including coronary heart disease and congestive heart failure (HF), are the leading cause of death worldwide. Obesity and diabetes are major risk factors for CVDs. About 2.6 billion people globally are overweight or obese, and 537 million adults were living with diabetes in 2021. In addition to inducing atherosclerosis, metabolic disorders can directly cause cardiac hypertrophy, fibrosis and cardiac diastolic and systolic dysfunctions, resulting in cardiomyopathy. However, the mechanisms underlying the pathogenesis of cardiomyopathy remain largely unclear.

Adipose tissue is an active metabolic organ that maintains the balance between lipid storage and energy utilization. Maladaptation of adipose tissue induces metabolic disorders, including insulin resistance, dyslipidemia, hepatic steatosis, and type 2 diabetes, which cause damage to multiple organs and contribute to the development of a variety of diseases 1-3. The heart has high energy demands, and its function is tightly associated with the health of adipose tissue 4, 5. Adipose tissue dysfunction, which occurs in both obesity and lipodystrophy, is recognized to have detrimental effects on the heart. Clinical investigations demonstrated that the incidence and prevalence of heart failure (HF) are high in obese patients, due to the direct adverse effects of obesity on left ventricular (LV) structure and function, and the increased risk of coronary heart disease. The pathological changes characterized by LV hypertrophy, cardiac fibrosis, diastolic and systolic dysfunction, and metabolic disorders originating from obesity alone are also known as obesity cardiomyopathy 6, 7. Interestingly, lipodystrophy manifested by generalized or partial loss of adipose tissue, can also cause cardiomyopathy, regardless of whether the etiology of lipodystrophy is congenital or acquired 8-10. Just like obesity cardiomyopathy, myocardial hypertrophy is the most prominent pathological change of lipodystrophy cardiomyopathy. Dilated cardiomyopathy and severe biventricular HF have also been described in patients with congenital lipodystrophy 8, 10.

Adipogenesis plays a central role in determining AT mass and functions. Recent studies revealed vacuolar H^+^-ATPase (V-ATPase) subunits ATP6AP2 and ATP6V0A1 regulates adipogenesis and adipose functions 11, 12. V-ATPase is highly conserved and expressed in virtually all eukaryotes as a multi-subunit complex, composed of a transmembrane V_o_ domain (subunits a, c, c', c'', d, e) that transports H^+^ across membrane, and a cytosolic V_1_ domain (subunits A-H) responsible for ATP hydrolysis. V-ATPase regulates the pH of intracellular vesicles, such as endosomes, lysosomes and Golgi apparatus 13-16, playing critical roles in receptor-mediated signaling, vesicle trafficking, receptor recycling, protein degradation and ion homeostasis 17-19. Beyond pumping protons across membranes, V-ATPase also acts as a central hub for monitoring and responding to changes in cellular nutrient levels and energy status by modulating the activity of mammalian target of rapamycin complex 1 and AMP activated kinase 20, 21. It is not yet clear whether other V-ATPase subunits play a role in regulating adipogenesis and AT functions. In the current study, we screened the expression of V-ATPase subunit during adipogenesis and the consequences of inhibiting V-ATPase subunits expression on adipogenesis using 3T3-L1 model. In this approach, we found that the expression of V_o_d1, which is encoded by Atp6v0d1, was increased as preadipocyte differentiates, while inhibiting its expression blocked differentiation of 3T3-L1 cells into mature adipocytes.

In the present study, we first identified V_o_d1 subunit as a master regulator of adipogenesis in 3T3-L1, and adipose-specific deletion of *Atp6v0d1* (*Atp6v0d1*^AKO^) in mice resulted in a complete loss of adipose tissues in adulthood. The *Atp6v0d1*^AKO^ mice spontaneously developed cardiac hypertrophy as early as at 12 weeks and progressed to HF at 28 weeks, resembling generalized lipodystrophy cardiomyopathy in patients. Using this animal model, we investigated the mechanisms underlying the pathogenesis of cardiomyopathy in *Atp6v0d1*^AKO^ mice. Here, we revealed a previously unrecognized role of myocardin in regulating cardiac insulin sensitivity and glucose and lipid metabolism through increasing the expression of insulin signaling/docking molecule insulin receptor substrate (IRS)-1. Myocardin was downregulated in the heart of *Atp6v0d1*^AKO^ mice, and restoring myocardin expression had a therapeutic effect against the development of cardiac hypertrophy and HF in *Atp6v0d1*^AKO^ mice.

## Methods

### Animal care

All animals were procured from the Animal Center of Guangdong Province, China, and were kept in specific pathogen-free conditions with unrestricted access to food and water. All animal procedures were sanctioned by the Institutional Care and Ethical Committee of Shenzhen University, China, and were performed in accordance with the Guide for the Care and Use of Laboratory Animals (National Institutes of Health publication No. 85-23, revised 1996) and the guidelines from Directive 2010/63/EU of the European Parliament on the protection of animals used for scientific purposes.

### Generation of the Atp6v0d1 Knockout (*Atp6v0d1*^AKO^) mice

*Atp6v0d1* floxed mice were generated using CRISPR/Cas9 system and provided by the Model Animal Research Center of Nanjing University (Nanjing, China). To genotype the floxed *Atp6v0d1* allele, forward primer (5'-GGCTACTGTGTGAGGAATGGAC-3') and reverse primer (5'-GGTTGGACTGCAGC-AAAGG-3') were used. *Atp6v0d1* floxed mice were crossed with Adipo-Cre mice (Jackson Laboratory) to generate adipose-specific *Atp6v0d1* knockout mice (Adipo-Cre^+/0^ *Atp6v0d1*^flox/flox^), denoted as *Atp6v0d1*^AKO^. Mice were housed with a 12-h light/12-h dark cycle and fed *ad libitum*. Blood samples were collected via submandibular bleeding after 6 h of fasting, and blood glucose levels were determined using a glucometer (Roche). To measure glucose tolerance and insulin tolerance, mice were fasted for 16 h and injected intraperitoneally with 1 g/kg body weight glucose or 0.75 U/kg body weight insulin. Blood samples were collected via the tail vein at 0, 15, 30, 60, 90, and 120 min after injection.

### Rosiglitazone and virus administration

Rosiglitazone (5 mg/kg/day) or the vehicle (0.5% sodium carboxymethyl cellulose) was administered to male control or *Atp6v0d1*^AKO^ mice (aged 16 weeks) by intragastric gavage every other day. At 16 weeks and 26 weeks of age, control or *Atp6v0d1*^AKO^ mice were administered AAV9-cTnt-myocardin or AAV9 vectors at a dose of 2.5×10^11^ viral genomes (vg)/mice via tail vein injection of at 16 weeks and 26 weeks of age. At 36 weeks of age, all mice were euthanized with an overdose of pentobarbital (150 mg/kg).

### Construction of shRNA lentiviral vector and lentivirus infection

To achieve knockdown of *Atp6v0d1* expression in 3T3-L1 cells, oligonucleotides against *Atp6v0d1* (5'-GCGTTCAATAGCTGAACTTGT-3') were synthesized and inserted into the shRNA expressing lentiviral vector PLVx-U6-ccdB-EF1a-copGFP by homologous recombination. Scramble shRNA (5'-CCTAAGGTTAAGTCGCCCTCT-3') that does not target any known coding genes was cloned into the lentiviral vector. To produce lentivirus, shRNA expressing lentiviral shRNA expressing vectors were co-transfected with the packaging plasmids (pPSPAX2 & pCMV-VSVG at 2:1) into HEK293T cells, and lentiviral particles were collected and titered as previously described 22. To transduce 3T3-L1 cells, collected lentivirus were added into culture medium at multiplicity of infection (MOI) of 2, together with 5 mg/mL polybrene.

### Staining of lipid droplets in differentiated 3T3-L1 cells

3T3-L1 preadipocytes were obtained from ZenBio (North Carolina, USA) and cultured in preadipocyte medium (ZenBio) at 37 °C under 5% CO_2_ in a humidified incubator. To induce differentiation into mature adipocytes, 3T3-L1 cells were cultured to 100% confluence (Day 0). Two days later (Day 2), the culture medium was replaced with differentiation medium (ZenBio) and cells were cultured for a further three days. Subsequently, the cells were cultured in adipocyte medium (ZenBio), which was refreshed daily until Day 11 when full differentiation into mature adipocytes was achieved. Lipid droplets were stained with oil red O (ORO) and LipidSpot^TM^ 610 as previously described 23. In brief, for ORO staining, differentiated 3T3-L1 cells were fixed with 4% PFA, rinsed twice with 60% isopropanol and then stained with ORO (Sigma, 0.6% in 60% isopropanol) for 30 min at room temperature. Stained samples were then rinsed twice with 60% isopropanol and then with PBS. For fluorescence staining, cells were fixed with 4% paraformaldehyde (PFA) and then permeabilized with 0.1% Triton X-100 for 10 min. The cells were incubated first with LipidSpot^TM^ 610 (1:1,000, Biotium) for 10 min followed by DAPI solution (Solarbio) for 5 min at room temperature, avoiding light exposure. Images were captured with the Cytation 5 Imaging Multimode Reader (Biotek).

### Hepatic lipid extraction and measurement

Hepatic lipids were extracted using Folch's method 24. Briefly, liver samples (50 mg) were homogenized with 625 μL chloroform/methanol mixture (2:1). After adding 125 μL chloroform, the homogenate was mixed and centrifuged at 14,000 ×*g* for 10 min at 4 ℃. The supernatant was transferred into a new Eppendorf (EP) tube and 250 μL methanol was added before the mixture was mixed again and centrifuged at 2,400 ×*g* for 5 min. Extracted lipids in the chloroform phase were collected and transferred into a new EP tube. The extracted lipids were then dried under nitrogen gas and resuspended in 100 μL PBS containing 1% Triton X-100 (Sigma-Aldrich). The levels of cholesterol, triglycerides, and non-esterified fatty acids (NEFAs) were measured using commercial kits (BioSino).

### Measurement of ALT and AST

Blood samples were pre-cleared by centrifugation at 3,000 ×*g* for 5 min at 4 ℃. Plasma alanine aminotransferase (ALT) and aspartate aminotransferase (AST) activities were measured using commercial kits (BioSino) following the manufacturer's instructions.

### H&E staining and ORO staining of tissue sections

H&E staining was performed on 4 μm sections prepared from tissues fixed with 4% PFA and embedded in paraffin. Neutral lipids were detected in ORO staining of 7 μm sections prepared from tissues embedded in optimal cutting temperature (OCT) compound. After fixation with 4% PFA, sections were stained with 0.3% ORO following standard procedures. Images were captured with the Cytation 5 Imaging Multimode Reader (Biotek).

### Isolation and culture of rat neonatal cardiomyocytes

Neonatal rat ventricular myocytes (NRVMs) were isolated and cultured according to previously described methods 25. In brief, Sprague-Dawley rats (aged 1-2 days) were humanely euthanized by isoflurane inhalation followed by cervical dislocation. The ventricles were then removed and digested in PBS solution containing 0.75 mg/ml collagenase (type II, Worthington, USA). After pre-plating the cells for 2 h, the supernatant containing purified cardiomyocytes was collected and cultured in DMEM supplemented with 10% fetal bovine serum (FBS, MIKX), 1% penicillin-streptomycin, and bromodeoxyuridine (1:100, to inhibit fibroblast growth) for an additional 48 h before further treatment.

### Isolation of adult mouse cardiomyocytes

Control and *Atp6v0d1*^AKO^ mice were anesthetized with pentobarbital sodium (50 mg/kg i.p.), and their hearts were quickly removed. The hearts were then subjected to retrograde aortic perfusion with Ca^2+^-free buffer [150 mM NaCl, 5.4 mM KCl, 1.2 mM MgSO4, 10 mM glucose, 5 mM HEPES, 10 mM 2,3-butanedione monoxime (BDM; Sigma), and 5 mM taurine (Sigma)] at a constant rate of 5-6 mL/min and a temperature of 37 °C for 2 min. Collagenase type II (1 mg/mL; Worthington) was then added to the perfusion solution to initiate enzymatic digestion. After 15 min, the ventricles were quickly removed, cut into several pieces, and further digested by gentle agitation for 3 min at 37 ℃ in the same enzyme solution. This process released single myocytes, which were collected in the supernatant and then pelleted by low-speed centrifugation.

### Echocardiography

Echocardiography was performed on non-anesthetized mice using a Vevo 2100 system (Visual Sonics, Toronto, Ontario, Canada), as previously described 26. For two-dimensional (2-D) guided M-mode echocardiography, the heart image was captured in the parasternal short-axis view. The following parameters were measured from this view: percentage of left ventricular (LV) fractional shortening (FS), LV ejection fraction (EF), LV internal dimensions at both diastole and systole (LVIDd and LVIDs, respectively), and LV posterior wall dimensions at both diastole and systole (LVPWd and LVPWs, respectively). LV fractional shortening (FS, %) was calculated as [(LVIDd - LVIDs)/LVIDd] × 100, and LV ejection fraction (EF, %) was calculated as [(LVIDd2 - LVIDs2)/ LVIDd2] × 100.

### Western blot analysis

Samples of mouse tissue or cells were homogenized in RIPA buffer (Solarbio, Beijing, China) supplemented with protease inhibitor cocktail (Cat #HY-K0010, MCE, Monmouth Junction, NJ, USA) and Phosphatase Inhibitor Cocktail II & III (Cat #HY-K0022& HY-K0023, MCE). Lysates were cleared by centrifugation at 14,000 ×*g* for 10 min at 4 ℃. The total protein concentration in the supernatant was determined by BCA assay (Pierce). Equal amounts of protein (20-50 μg in total) were separated by SDS-PAGE and then transferred to PVDF membranes (Merck Millipore, Bedford, MA, USA). The blots were then probed with the antibodies listed in **Supplementary Table 1,** detected by Western Chemiluminescent HRP Substrate (Merck Millipore, Bedford, MA) and visualized using a UVP ChemStudio PLUS imaging system (CA, USA). Band intensities were quantified using ImageJ (NIH, USA).

### Immunofluorescence

Control and *Atp6v0d1*^AKO^ hearts were fixed in 4% paraformaldehyde, embedded in paraffin, and sectioned at 5 μm intervals. For antigen retrieval, the sections were dehydrated in a graded series of ethanol solutions and then microwaved in citrate buffer (0.4 g/L citric acid and 3 g/L sodium citrate) for 3 min. Permeabilization and blocking were performed using 5% BSA (IgG free) and 0.2% Triton X-100 in PBS at room temperature for 30 min. The sections were then incubated overnight at 4 ℃ with primary antibodies. The samples were further incubated with corresponding secondary antibodies and visualized by adding 4′,6-Diamidine-2′-phenylindole dihydrochloride (DAPI, Cat #10236276001, Sigma-Aldrich, Burlington, MA, USA). Confocal images were captured using a Zeiss LSM880 microscope (Carl Zeiss, Germany).

### Real-time quantitative PCR

Total RNA from heart tissue and cell samples was extracted using TRIzol reagent (Invitrogen, Carlsbad, CA, USA) according to the standard protocol. Genomic DNA contamination was removed from total RNA (500 ng-1 µg) using gDNA wiper mix (Vazyme, Nanjing, China), followed by reverse transcription using HiScript III qRT Supermix (Vazyme, Nanjing, China) according to the manufacturer's instructions. Quantitative real-time PCR (qPCR) was performed with ChamQ Universal SYBR qPCR master mix and gene-specific primers on a QuantStudio 3 real-time PCR System (Applied Biosystems, Waltham, MA, USA). Data were normalized against the GAPDH gene and the relative expression level of each gene was calculated using the 2-∆∆Ct method. Primers used in the study are listed in **Supplementary Table 2**.

### Oroboros O2K measurement

The oxygen concentration and oxygen flux of intact cells were measured using the Oxygraph-2k (O2k, OROBOROS Instruments, Innsbruck, Austria) according to the manufacturer's instructions 27. Experiments were conducted using isolated adult cardiomyocytes in culture medium (10^6^ cells/mL) and performed in two O2k chambers maintained at 37 ℃. The oxygen consumption rate (OCR) was measured under basal conditions and in response to oligomycin (oligo), FCCP, and rotenone plus antimycin A (Rot/AM). Basal OCR was measured before oligomycin injection and maximum OCR was measured after FCCP injection and the non-mitochondrial respiration (measurement after rotenone and antimycin A injection) was subtracted.

### Small interfering RNA-mediated gene knockdown

Small interfering RNA (siRNA) and scrambled siRNA were chemically synthesized by Ribobio (Guangzhou, China). The designed siRNA sense sequences were as follows: si-Myocd, 5′-CCTGGTTAATATGCACATT-3′; si-Irs1, 5′-GAGAAGAAGTGGC-GGCACA-3′. Cells were transfected with of siRNA (30 pmol/L) using Lipofectamine™ RNAiMAX Transfection Reagent (Invitrogen, Carlsbad, CA, USA) according to the manufacturer's instructions and cultured for 48 h. The medium was then changed and the culture was continued for a further 24 h before collection for total RNA.

### Reporter plasmids and luciferase assays

The IRS1 promoter, cloned from rat heart chromatin, was inserted between the *Nhe*I and *Hind*III restriction enzyme sites into the Promega pGL4.10 promoterless luciferase vector, resulting in the construction of the pGL4.10-IRS1 vector. For dual luciferase reporter assay, HEK293T cells were transiently transfected with Lipofectamine 3000 reagent (Thermo Fisher Scientific) according to the manufacturer's instructions. Briefly, cells were seeded into 24-well plates, and cultured to 80% confluence before transfection with either 500 ng pGL4.10 promoterless control or pGL4.10-IRS1 and 500 ng SRF-expressing plasmid or pcDNA3.1. Cells were co-transfected with Renilla plasmid as an internal control. After transfection, cells were cultured for an additional 24 h and lysed for the measurement of luciferase and Renilla activity using the TransDetect® Double-Luciferase Reporter Assay Kit (TransGen) according to the manufacturer's instructions.

### Chromatin immunoprecipitation

For chromatin immunoprecipitation (ChIP) assays, The Magna ChIP A/G Chromatin Immunoprecipitation Kit (Millipore Sigma) was utilized according to the manufacturer's instructions. Briefly, NRVM were fixed with 1% formaldehyde for 10 min at room temperature and then quenched with glycine for 5 min at room temperature. After sonication, equal amounts of cell lysate were immunoprecipitated with either anti-SRF antibody or IgG (#2729, Cell Signaling Technology, Boston, MA) at 4 ℃ overnight. The DNA fragments were eluted from the protein/DNA complexes by routine washing steps and then analyzed by quantitative PCR using the primers listed in the **Supplementary Table 2**. The IRS1 promoter region flanking binding sites served as target regions, and the distal regions located approximately 1 kb and 2 kb from the transcription start site served as control regions.

### RNA-sequencing and bioinformatics

Total RNA was isolated from heart tissues of 36-week-old control and *Atp6v0d1*^AKO^ mice. The quality and integrity of total RNA were evaluated using the Agilent Technologies 2100 Bioanalyzer. RNA-Seq libraries were sequenced on the Illumina NovaSeq 6000. The level of mRNA expression was normalized using FPKM (fragments per kilobase of transcript per million mapped reads). Clean reads were mapped to the reference gene using featureCounts (v1.5.0-p3), and to the reference genome using Hisat2 (v2.0.5). Differential expression analysis was performed using the DESeq2 R package (1.20.0). The resulting *P*-values were adjusted using the Benjamini and Hochberg approach to control for the false discovery rate. The threshold for significantly differential expression was set as *P*adj < 0.05 and |log2(foldchange)| > 0.5. Gene Ontology (GO) enrichment analysis of the differentially expressed genes was performed using the clusterProfiler R package (3.8.1), with enrichment declared for corrected *P* < 0.05. Reactome, an open-source, curated and peer-reviewed pathway database (https://reactome.org/) 28, was used to perform pathways enrichment on significant gene sets.

### Statistical analysis

All results are analyzed using GraphPad Prism software and presented as the means ± SEM. Unpaired t-test and one-way ANOVA were used for two-group or multiple-group comparisons. The details of statistical analysis for figures and Supplementary Figures are performed as indicated in the figure legends.

## Results

### *Atp6v0d1* regulates adipogenesis and its loss leads to lipodystrophy

We found an increase in *Atp6v0d1* expression during differentiation of 3T3-L1 preadipocytes into mature adipocytes (Figure S1A and S1E), in parallel with elevated expression of *Pparg*, *Cebpa*, and *Cebpb*, which are markers of adipogenesis (Figure S1B-S1D). Silencing *Atp6v0d1* expression led to downregulation of PPARγ, CEBPα and FASN, along with the reduced lipid droplet accumulation, an indicator of mature adipocytes, in 3T3-L1 adipocytes (Figure S1F and S1G), suggesting ATP6V0D1 as a master regulator of adipogenesis. Moreover, analysis of adipose tissues from diet-induced-obesity mice revealed a marked increase in ATP6V0D1 abundance (Figure S1H and S1I), suggesting V_o_d1 subunit is involved in maintaining adipose homeostasis.

**Figure 1:**
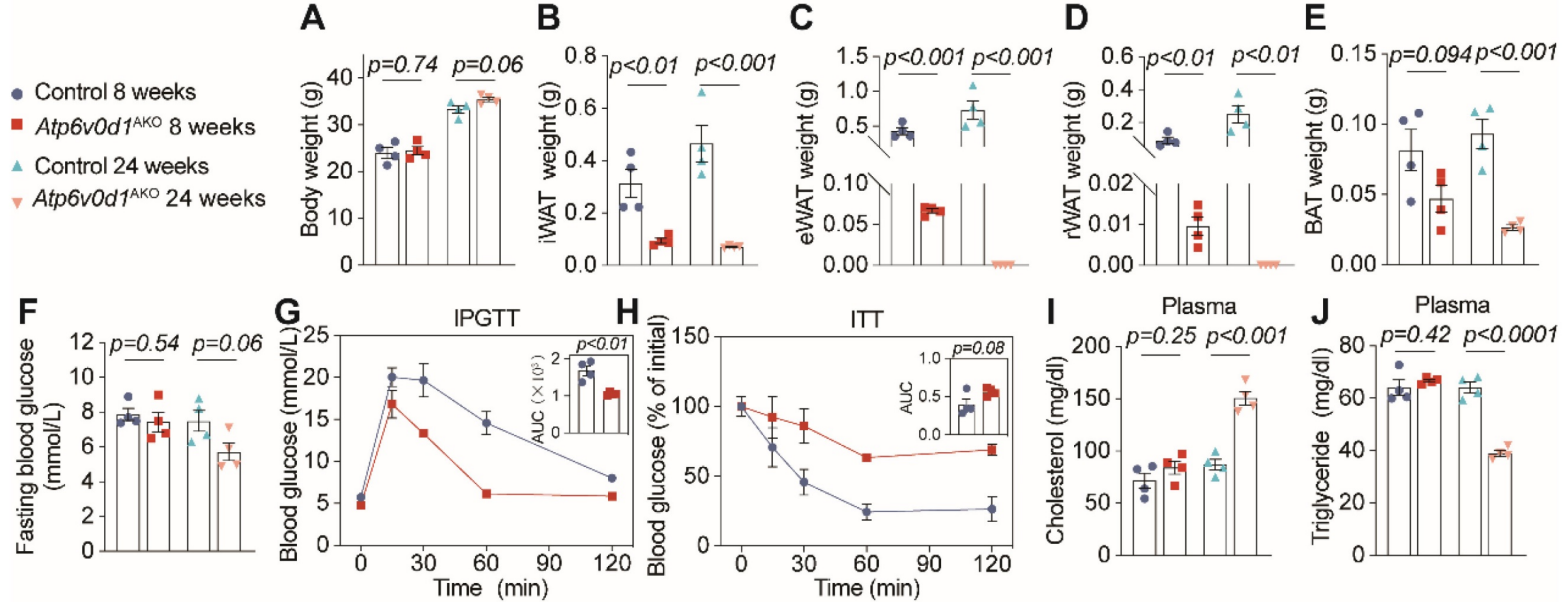
***Atp6v0d1^AKO^* mice exhibit lipodystrophy and insulin resistance. A**, Body weight. **B**, Weights of inguinal WAT (iWAT). **C**, Epididymal white adipose tissue (eWAT). **D**, Retroperitoneal WAT (rWAT). **E**, Brown adipose tissue (BAT). **F**, Fasting blood glucose. **G**, Intraperitoneal glucose tolerance test (IPGTT) comparisons using area under curve (AUC). **H**, Insulin tolerance test comparisons using AUC. **I**, Plasma cholesterol. **J**, Plasma triglyceride. Data represent the mean ± SEM (n = 4) (two-tailed unpaired Student's *t*-test).

We further generated adipocyte-specific *Atp6v0d1* knockout mice, denoted as *Atp6v0d1*^AKO^, by mating *Atp6v0d1* floxed mice with Adiponectin-Cre mice (Figure S2A-S2C). Despite unaltered body weight (Figure 1A and Figure S2D), *Atp6v0d1*^AKO^ mice exhibited markedly diminished adipose depots compared to their littermate controls at both 8 and 24 weeks of age (Figure 1B-1E and Figure S2E-S2F). Notably, at 24 weeks of age, *Atp6v0d1*^AKO^ mice even had no visible epididymal and retroperitoneal white adipose depots, accompanied by reduction in plasma leptin and adiponectin levels (Figure S2G and S2H). These data suggested that ATP6V0D1 deficiency in adipocytes led to a progressive loss of adipose depots, likely due to the disruption of adipogenesis. Consistent with this, hepatic steatosis emerged at 8 weeks of age in *Atp6v0d1*^AKO^ mice (Figure S2I-S2L), progressing to liver injury as measured by AST to ALT levels at 24 weeks (Figure S2M). Furthermore, 24-week-old *Atp6v0d1*^AKO^ mice displayed significant disturbances of glucose and lipid metabolism, manifested as increased glucose tolerance but reduced insulin sensitivity (Figure 1F-H), increased plasma cholesterol (Figure 1I), and reduced triglycerides levels (Figure 1J). The plasma cholesterol remained high, and high NEFA levels and systemic insulin resistance were also observed in *Atp6v0d1*^AKO^ mice aged 36 weeks (Figure S3A-S3E).

### Adipocyte ATP6V0D1 deficiency leads to cardiac hypertrophy and contractile dysfunction

**Figure 2:**
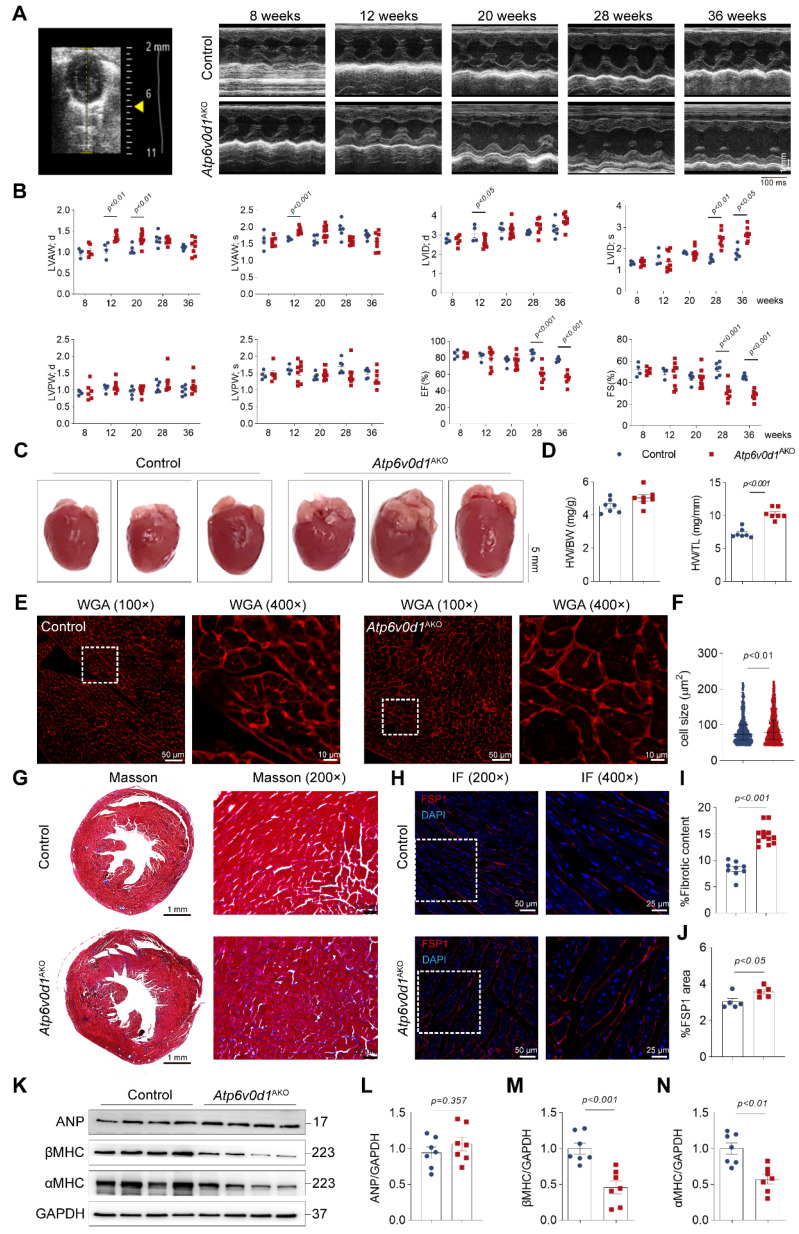
***Atp6v0d1*^AKO^ mice exhibit cardiac hypertrophy and fibrosis. A**, Representative M-mode tracings from the echocardiography of control and *Atp6v0d1*^AKO^ mice aged 8, 12, 20, 28 and 36 weeks. **B**, Echocardiographic parameters recorded in (A) (n = 4-8). LVAWs, thickness of left ventricular anterior wall at systole; LVAWd, thickness of left ventricular anterior wall at diastole; LVPWs, thickness of left ventricular posterior wall at systole; LVPWd, thickness of left ventricular posterior wall at diastole; LVIDs, systolic left ventricular internal dimension; LVIDd, diastolic left ventricular internal dimension; EF left ventricular ejection fraction; FS, fractional shortening. **C**, Representative images of hearts from control and *Atp6v0d1*^AKO^ mice aged 36 weeks. **D**, HW/BW and HW/TL between control and *Atp6v0d1*^AKO^ mice. HW, heart weight; BW, body weight; TL, tibial length. **E**, Representative images of WGA stain from control and *Atp6v0d1*^AKO^ mice aged 36 weeks. **F**, Quantification of the cell size for each group. **G**, Representative transverse sections of control and *Atp6v0d1*^AKO^ hearts with Masson trichrome staining. **H**, Immuno-fluorescence imaging of FSP1. Control and *Atp6v0d1*^AKO^ heart sections showing red signal for anti-FSP1 antibody. Cell nuclei are stained with DAPI (blue). **I**, Quantification of fibrotic content in control and *Atp6v0d1*^AKO^ heart sections. **J**, % Fluorescent area for FSP1 area in control and *Atp6v0d1*^AKO^ heart sections. **K-N**, Representative immunoblots and quantification of α-MHC, β-MHC and ANP protein levels in control and *Atp6v0d1*^AKO^ heart tissues. Data are normalized to GAPDH and analyzed by two-tailed unpaired Student's *t*-test. Data represent the mean ± SEM (n = 7).

We then monitored the cardiac structure and function in *Atp6v0d1*^AKO^ mice and control mice (*Atp6v0d1*^flox/flox^ littermates) with echocardiography. At 8 weeks, the thickness of the anterior and posterior walls of the left ventricle (LVAW and LVPW, respectively) at systole (s) and diastole (d), the internal LV diameters at systole (LVIDs) and diastole (LVIDd), and the LV ejection fraction (EF%) and fractional shortening (FS%) were comparable in control and *Atp6v0d1*^AKO^ mice (Figure 2A-2B and Figure S4A). At 12 weeks, the LVAWs and LVAWd in *Atp6v0d1*^AKO^ mice were significantly larger than those in control (Figure 2B). In contrast, the LVIDd in *Atp6v0d1*^AKO^ mice was smaller than that in their control littermates (Figure 2B). The EF% and FS% were comparable in these two groups of mice until 24 weeks (Figure 2B and Figure S4B-S4C), suggesting that the *Atp6v0d1*^AKO^ mice developed cardiac hypertrophy with preserved contractile function. At 28 weeks, apparent cardiac contractile dysfunction was observed in *Atp6v0d1*^AKO^ mice as evidenced by significantly larger LVIDs, and remarkedly reduced EF% and FS% (59.4% and 31.2%, respectively, in *Atp6v0d1*^AKO^ mice versus 84.3% and 52.6%, respectively, in control mice), indicating the development of HF (Figure 2A-2B). There was only a minor and insignificant decrease in EF% and FS% in *Atp6v0d1*^AKO^ mice aged 36 weeks compared with those in *Atp6v0d1*^AKO^ mice aged 28 weeks (Figure 2A-2B), suggesting the progress of HF is slow. The hearts of *Atp6v0d1*^AKO^ mice aged 36 weeks were visibly larger than those of their litter controls (Figure 2C). However, there was no difference in the HW to body weight (BW) ratio (HW/BW) between *Atp6v0d1*^AKO^ mice and their littermate controls (Figure 2D); however, the significantly greater weight of the liver in *Atp6v0d1*^AKO^ mice indicated that HW/BW ratio may not be suitable to reflect cardiac hypertrophy (Figure S3F). Therefore, we analyzed the ratio of HW to tibia length (HW/TL), and found that HW/TL in *Atp6v0d1*^AKO^ mice was much larger than that of the littermate controls (Figure 2D). Histological images also showed that the heart size in *Atp6v0d1*^AKO^ mice was larger than that in control mice (Figure 2C). WGA staining of the heart slices showed a significant increase in the size of cardiomyocytes in *Atp6v0d1*^AKO^ mice compared to control mice (Figure 2E and 2F). These results further confirmed cardiac hypertrophy in *Atp6v0d1*^AKO^ mice. Moreover, Masson Trichrome staining revealed that fibrosis occurred in *Atp6v0d1*^AKO^ hearts (Figure 2G and 2I). Positive areas for immunostaining of FSP, the biomarker for fibroblasts, were also increased in *Atp6v0d1*^AKO^ hearts (Figure 2H and 2J), suggesting enhanced proliferation of cardiac fibroblasts. Intriguingly, despite the apparent cardiac dysfunction in *Atp6v0d1*^AKO^ mice at 36 weeks, there was no difference in the myocardial protein abundance of ANP, a fetal gene that is activated in HF, compared with control mice, while α- and β-MHC proteins were both significantly decreased (Figure 2K-2N). Furthermore, the mRNA levels of *Nppa* and *Nppb*, which encode ANP and BNP, respectively, were consistent with those of the corresponding proteins, while the mRNA levels of *Myh6* and *Myh7*, which encode α-MHC and β-MHC, respectively, were significantly decreased in *Atp6v0d1*^AKO^ hearts (Figure S4D). These data indicated that *Atp6v0d1*^AKO^ mice develop cardiac hypertrophy and HF with relatively low ANP expression.

### Excessive lipid accumulation and reprogramed energy expenditure occur in *Atp6v0d1*^AKO^ hearts

**Figure 3:**
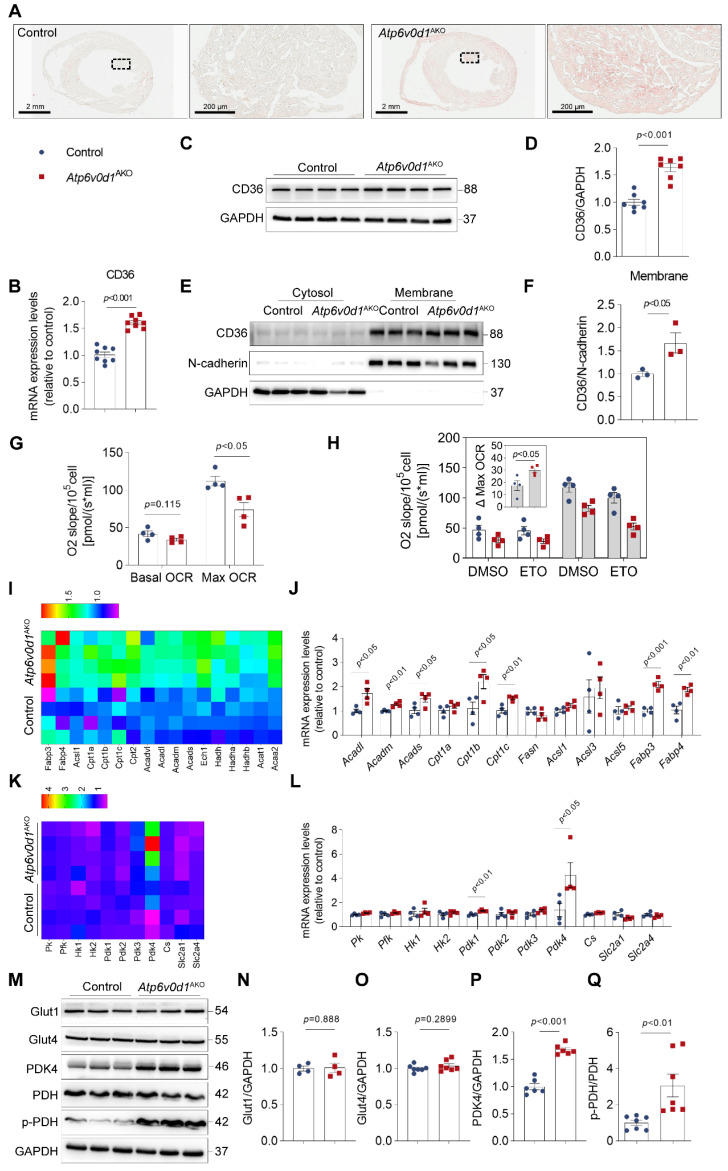
** Excessive lipid is accumulated and the energy expenditure is reprogramed in *Atp6v0d1*^AKO^ hearts. A**, Representative images of oil red O staining of sections of control and *Atp6v0d1*^AKO^ hearts. **B**, qPCR quantification of CD36 mRNA levels in control and *Atp6v0d1*^AKO^ heart tissues. **C-D**, Representative immunoblots and quantification of CD36 protein levels in control and *Atp6v0d1*^AKO^ heart tissues. **E**-**F**, Representative immunoblots and quantification of cell-surface CD36 in heart lysates from both control and *Atp6v0d1*^AKO^ heart tissues. **G**, Quantification of basal and maximal OCR levels of isolated cardiomyocytes from control and *Atp6v0d1*^AKO^ hearts. **H**, Quantification of Eto-sensitive OCR levels of isolated cardiomyocytes from control and *Atp6v0d1*^AKO^ hearts. OCR was measured using the Oroboros O2k with the addition of etomoxir (Eto) at the baseline. **I**, Heatmap analysis of fatty acid oxidation (FAO)-involved genes from the RNA-sequencing dataset of control and *Atp6v0d1*^AKO^ hearts. **J**, qPCR quantification of the mRNA levels of FAO-related genes in control and *Atp6v0d1*^AKO^ hearts. **K**, Heatmap analysis of glycolysis and glucose oxidation-related genes from the RNA-sequencing dataset of control and *Atp6v0d1*^AKO^ hearts. **L**, qPCR quantification of the mRNA levels of glycolysis and glucose oxidation-related genes in control and *Atp6v0d1*^AKO^ hearts. **M**, Representative immunoblots of GLUT1, GLUT4, PDK4, PDH, p-PDH protein levels in control and *Atp6v0d1*^AKO^ heart tissues. **N-Q**, Quantification of specified densitometric ratios. Data represent the mean ± SEM (n = 4-7) (two-tailed unpaired Student's *t*-test).

Oil red O (ORO) staining of heart cryosections showed excessive lipid deposition in *Atp6v0d1*^AKO^ hearts (Figure 3A). In accordance with this finding, the mRNA, total protein levels and cell-surface expression of cluster of differentiation 36 (CD36), the major transporter of cardiac FA uptake, were significantly increased in *Atp6v0d1*^AKO^ hearts compared with the controls (Figure 3B-3F), suggesting CD36 upregulation likely contributes to lipid infiltration in *Atp6v0d1*^AKO^ hearts. We further mapped the oxidative respiratory status and capacity by measuring oxygen consumption rate (OCR) and fuel dependency using isolated cardiomyocytes from control and* Atp6v0d1*^AKO^ mice. Basal OCR levels of isolated *Atp6v0d1*^AKO^ cardiomyocytes were comparable to those of their littermate controls (Figure 3G and Figure S5A). However, FCCP (Trifluoromethoxy carbonylcyanide phenylhydrazone)-induced maximal respiration was reduced in *Atp6v0d1*^AKO^ cardiomyocytes (Figure 3G), despite the comparable abundance of the mitochondrial respiratory complexes between hearts of *Atp6v0d1*^AKO^ and control mice (Figure S5B). Following application of etomoxir, an inhibitor for carnitine palmitoyl-transferase (CPT1) that is responsible for importing long chain fatty acid into mitochondria for oxidation, the reduction in FCCP-induced maximal respiration was greater in *Atp6v0d1*^AKO^ cardiomyocytes than that in controls (Figure 3H). The results suggested that the amount of FAO-supplied energy is increased in *Atp6v0d1*^AKO^ cardiomyocytes, despite a reduction in maximal respiratory capacity.

To understand the molecular mechanisms underlying the abnormal lipid metabolism and the development of hypertrophic cardiomyopathy, we examined the RNA expression profiles obtained from unbiased RNA sequencing (RNA-seq). Using |Log_2_FC|>0.5 and P_adjusted_<0.05 as cutoff values, we identified 527 upregulated genes and 262 downregulated genes (Figure S6A). GO and Reactome enrichment analysis indicates that lipid metabolism pathway and fatty acid metabolism pathway were among the top 15 most affected pathways in *Atp6v0d1*^AKO^ hearts (Figure S6B and S6C). In accordance with the FAO shift, transcriptomic profiles revealed that FAO-related genes, including *Acadl*, *Acadm*, *Acads*, *Cpt1b*, *Cpt1c*, *Acsl1*, *Fabp3* and *Fabp4*, were significantly upregulated in *Atp6v0d1*^AKO^ hearts compared with control hearts (Figure 3I), which were confirmed by quantitative PCR (Figure 3J).

Lipid and glucose metabolism are competitive processed in terms of heart energy expenditure as described by the glucose fatty-acid cycle (Randle cycle) 29. The increase in the utilization of FA as an energy source suggested a reciprocal repression of glucose uptake and oxidation in *Atp6v0d1*^AKO^ hearts. We found that there were no differences in the mRNA and protein levels of the cardiac glucose transporters Glut1 and Glut4 (encoded by *slc2a1* and *slc2a4*, respectively) between *Atp6v0d1*^AKO^ and control hearts (Figure 3L-3O). However, RNA-seq analysis, qPCR and Western blot assays identified a critical gene in glucose oxidation cascade, pyruvate dehydrogenase kinase 4 (PDK4) that was remarkably increased in *Atp6v0d1*^AKO^ compared with control hearts (Figure 3K-3M and 3P). PDK4 negatively regulates glucose oxidation by phosphorylation of pyruvate dehydrogenase (PDH), a rate-limiting enzyme that catalyzes oxidative decarboxylation of pyruvic acid to acetyl-CoA. Accordingly, we found the protein levels of phosphorylated PDH (p-PDH) rather than the total PDH were increased in *Atp6v0d1*^AKO^ hearts (Figure 3M and 3Q). The results indicated that PDK4 downregulation may lead to decreased glucose oxidation by phosphorylating PDH in *Atp6v0d1*^AKO^ mouse hearts.

### *Atp6v0d1*^AKO^ hearts exhibit insulin resistance

**Figure 4:**
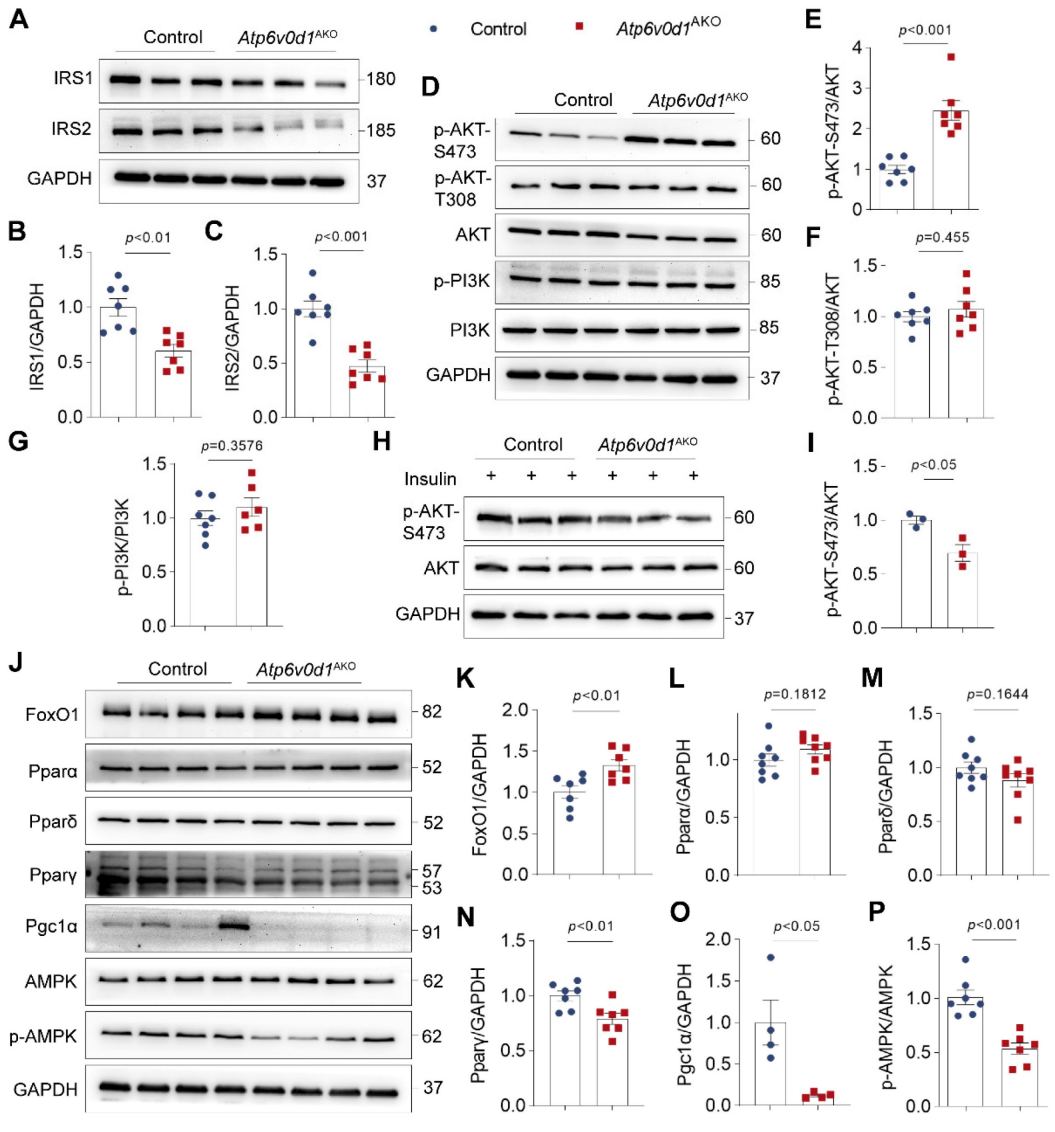
***Atp6v0d1*^AKO^ hearts exhibit insulin resistance. A-C**, Representative immunoblots and quantification of IRS1 and IRS2 protein levels. Data are normalized to GAPDH. Data represent the mean ± SEM (n = 7) (two-tailed unpaired Student's t-test). **D**, Total protein extracts from control and *Atp6v0d1*^AKO^ hearts immunoblotted with the indicated antibodies, representative immunoblots of three independent experiments are shown. **E-G**, Statistical analysis of specified densitometric ratios. Data represent the mean ± SEM (n = 7) (two-tailed unpaired Student's t-test). H-I, Immunoblot analysis and quantification of AKT phosphorylation at Ser473 phosphorylation in heart tissues from control and *Atp6v0d1*^AKO^ mice at 15 min after injection of insulin at 1 U/kg. Data represent the mean ± SEM (n = 3) (two-tailed unpaired Student's *t*-test). J, Total protein extracts from control and *Atp6v0d1*^AKO^ hearts immunoblotted with the indicated antibodies, representative immunoblots of four independent experiments is shown. K-P, Statistical analysis of specified densitometric ratios. Data represent the mean ± SEM (n = 4 - 7) (two-tailed unpaired Student's t-test).

To gain a better understanding of the molecular aspects of the changes in glucose and lipid metabolism, we examined the insulin signal activity, and the protein abundance of Forkhead box O1 (FoxO1), PPARs (PPARα, PPARδ and PPARγ), and AMPK, which play critical roles in regulating cardiac lipid and glucose metabolism 30-32, in the hearts of control and *Atp6v0d1*^AKO^ mice. The expression of insulin signaling/docking molecule insulin receptor substrate (IRS)-1 and IRS-2 were significantly decreased in *Atp6v0d1*^AKO^ heart compared to that in control mice (Figure 4A-4C), suggesting *Atp6v0d1*^AKO^ hearts developed insulin resistance. Insulin resistance has been shown to suppress PI3K-Akt signaling pathway 33. However, we found PI3K activity indicated by the phosphorylation levels of PI3K (p-PI3K) and Akt-T308 was not changed (Figure 4D-4G), while the protein level of Akt phosphorylated at Ser473 (p-Akt-S473) and the p-Akt-S473/Akt ratio was remarkably increased in *Atp6v0d1*^AKO^ hearts compared with the control (Figure 4D and 4E). In addition to insulin signal, PI3K-Akt activity can be regulated by multiple other signaling pathways. We thus examined PI3K-Akt activity in control and *Atp6v0d1*^AKO^ hearts in response to insulin stimulation. The results showed that the protein level of phosphorylated AKT-S473 was significantly decreased, confirming insulin resistance in *Atp6v0d1*^AKO^ hearts (Figure 4H-4I).

The protein abundance of FoxO1 was significantly increased (Figure 4J and 4K). There were no significant differences in the protein levels of PPARα and PPARδ, which are highly expressed in heart and play critical role in regulating lipid metabolism, between *Atp6v0d1*^AKO^ and control hearts (Figure 4L and 4M), but the protein abundances of PPAR-γ and PGC-1, the PPARγ coactivator-1, which are important in lipogenesis and lipid synthesis in white adipose tissue, were significantly decreased in *Atp6v0d1*^AKO^ relative to control hearts (Figure 4N and 4O). Moreover, the phosphorylated AMPK (p-AMPK) were significantly decreased, while the total AMPK expression was unchanged in *Atp6v0d1*^AKO^ relative to control hearts (Figure 4P).

### Increasing myocardin expression reverses cardiac insulin resistance and metabolic disorder, improving cardiac functions in *Atp6v0d1*^AKO^ mice

**Figure 5:**
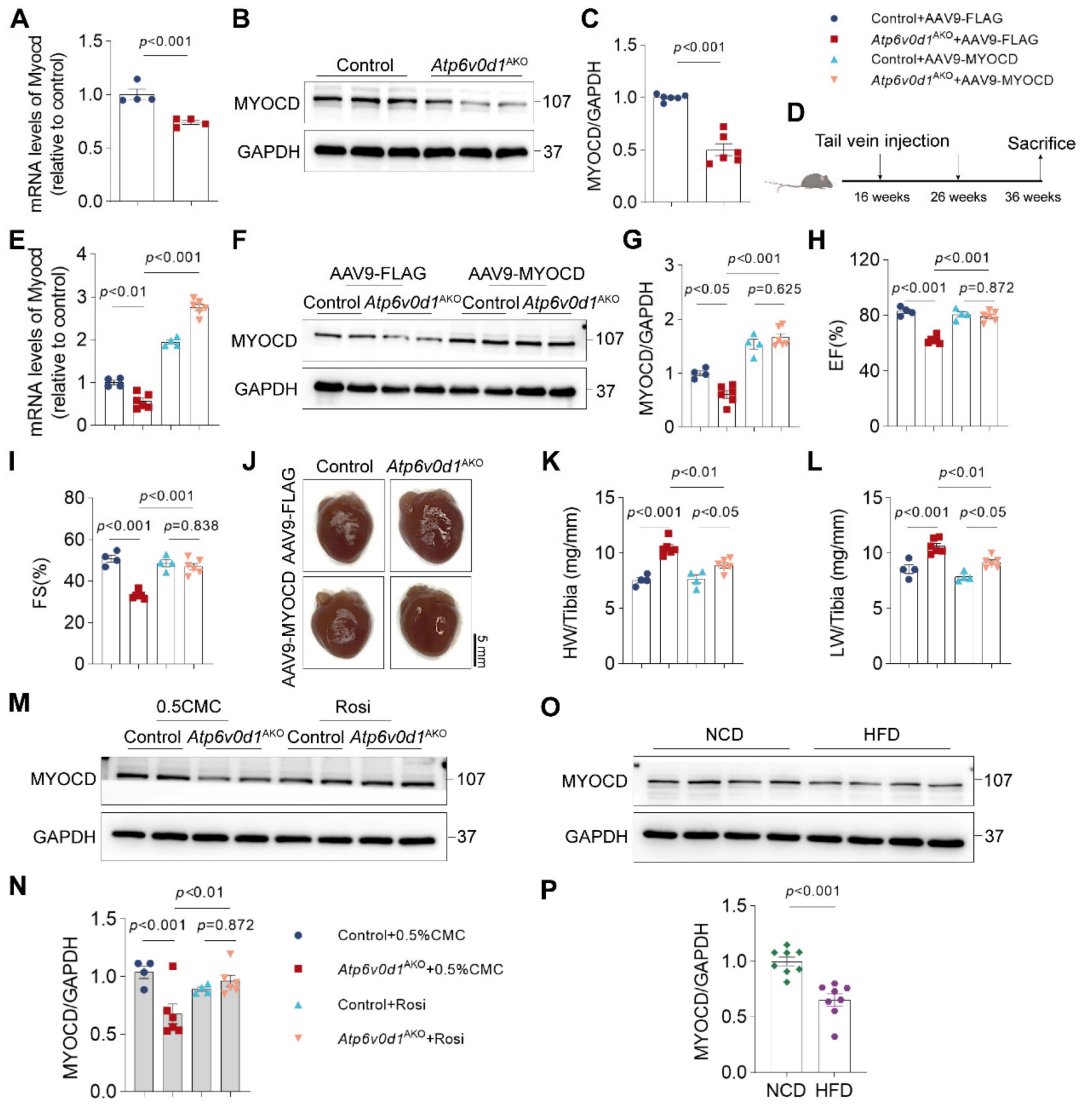
** Increased myocardin expression ameliorates cardiac dysfunction in *Atp6v0d1*^AKO^ mice. A**, qPCR quantification of the mRNA levels of myocardin in control and *Atp6v0d1*^AKO^ hearts. Data represent the mean ± SEM (n = 4) (two-tailed unpaired Student's t-test). **B-C**, Representative immunoblots and quantification of myocardin protein levels in control and *Atp6v0d1*^AKO^ heart tissues. Data represent the mean ± SEM (n = 6) (two-tailed unpaired Student's *t*-test). **D**, Schematic illustration of AAV-mediated gene therapy, delivered via intravenous injection to control and *Atp6v0d1*^AKO^ mice. **E**, qPCR quantification of the mRNA levels of myocardin. Data represent the mean ± SEM (n = 4 - 6) (one-way ANOVA). **F**, AAV9-mediated expression of myocardin in control and *Atp6v0d1*^AKO^ hearts. **G**, Quantification of myocardin protein levels. Data represent the mean ± SEM (n = 4 - 6) (one-way ANOVA). **H-I**, Echo measurement of left ejection fraction (EF%) and fractional shortening (FS%) of control and *Atp6v0d1*^AKO^ mice after tail vein injection of AAV9-cTnt-myocardin or AAV9-vectors. Data represent the mean ± SEM (n = 4 - 6) (one-way ANOVA). **J**, Representative images of hearts from control and *Atp6v0d1*^AKO^ mice after tail vein injection of AAV9-cTnt-myocardin or AAV9-vectors. **K-L**, Heart weight (HW) to tibia ratio and lung weight (LW) to tibia ratio of control and *Atp6v0d1*^AKO^ mice conducted with tail vein injection of AAV9-cTnt-myocardin or AAV9-vectors. Data represent the mean ± SEM (n = 4 - 6) (one-way ANOVA). **M-N**, Representative immunoblots and quantification of myocardin protein levels. Data represent the mean ± SEM (n = 4 - 6) (one-way ANOVA). **O-P**, Representative immunoblots and quantification of myocardin protein levels in normal chow diet (NCD) and high fat diet (HFD) mice. Data represent the mean ± SEM (n = 8) (two-tailed unpaired Student's t-test).

Through further analysis of the RNA-seq data, we found that myocardin expression was remarkably reduced in *Atp6v0d1*^AKO^ hearts. Quantitative PCR and Western blot analyses confirmed the remarkably decreased expression of myocardin in *Atp6v0d1*^AKO^ hearts (Figure 5A-5C). Myocardin is a transcription cofactor that plays an essential role in regulating cardiomyocyte survival and maintaining cardiac functions by interacting with serum response factor (SRF) to control the transcription of multiple genes containing cis element CArG boxes 34. To explore the possible role of myocardin downregulation in the pathogenesis of cardiomyopathy in *Atp6v0d1*^AKO^, we restored cardiac myocardin expression by tail-vein injection of AAV9 virus that expresses myocardin under the control of the cTNT promoter (AAV9-cTnt-Myocd) to *Atp6v0d1*^AKO^ mice aged 16 weeks (Figure 5D). Subsequently, we detected 1.9- and 1.85-fold increases in myocardin protein levels in the hearts of control and *Atp6v0d1*^AKO^ mice aged 36 weeks, respectively (Figure 5F-5G).

Although previous studies demonstrated that myocardin overexpression in cultured neonatal cardiomyocytes induced cardiomyocyte hypertrophy 35, we found that increasing myocardin expression level to this extent in adult mouse hearts had no significant effect on cardiac function and heart size, with comparable EF%, FS% and HW/TL in the control and myocardin-overexpressed (control + myocd) mice (Figure 5H-5L). However, restoring myocardin expression in the hearts of *Atp6v0d1*^AKO^ mice (*Atp6v0d1*^AKO^ + myocd) increased EF% and FS% to levels that were comparable to those in their littermate controls (Figure 5H-5I and Figure S8A), suggesting that the cardiac contractile functions of *Atp6v0d1*^AKO^ mice were completely restored. Cardiac hypertrophy, as measured by HW/TL, was also attenuated by increasing myocardin expression (Figure 5J-5L). In addition, restoring myocardin expression in the hearts of *Atp6v0d1*^AKO^ mice also reduced cardiomyocyte size and cardiac fibrosis (Figure S8B-S8D). These results collectively indicated that myocardin downregulation is critical for the onset and development of cardiomyopathy in *Atp6v0d1*^AKO^ mice. Interestingly, we found that treatment with Rosiglitazone, a systemic insulin sensitizer which improved cardiac metabolism and function in *Atp6v0d1*^AKO^ mice (Figure S7), completely restored myocardin expression in *Atp6v0d1*^AKO^ hearts (Figure 5M-5N), suggesting a causal link between systemic insulin resistance and myocardin downregulation. In hearts of high fat diet-induced type 2 diabetes mice, we also found the expression of myocardin, as well as IRS1, was decreased (Figure 5O-5P and Figure S8E-S8F), providing further evidence for this hypothesis.

**Figure 6:**
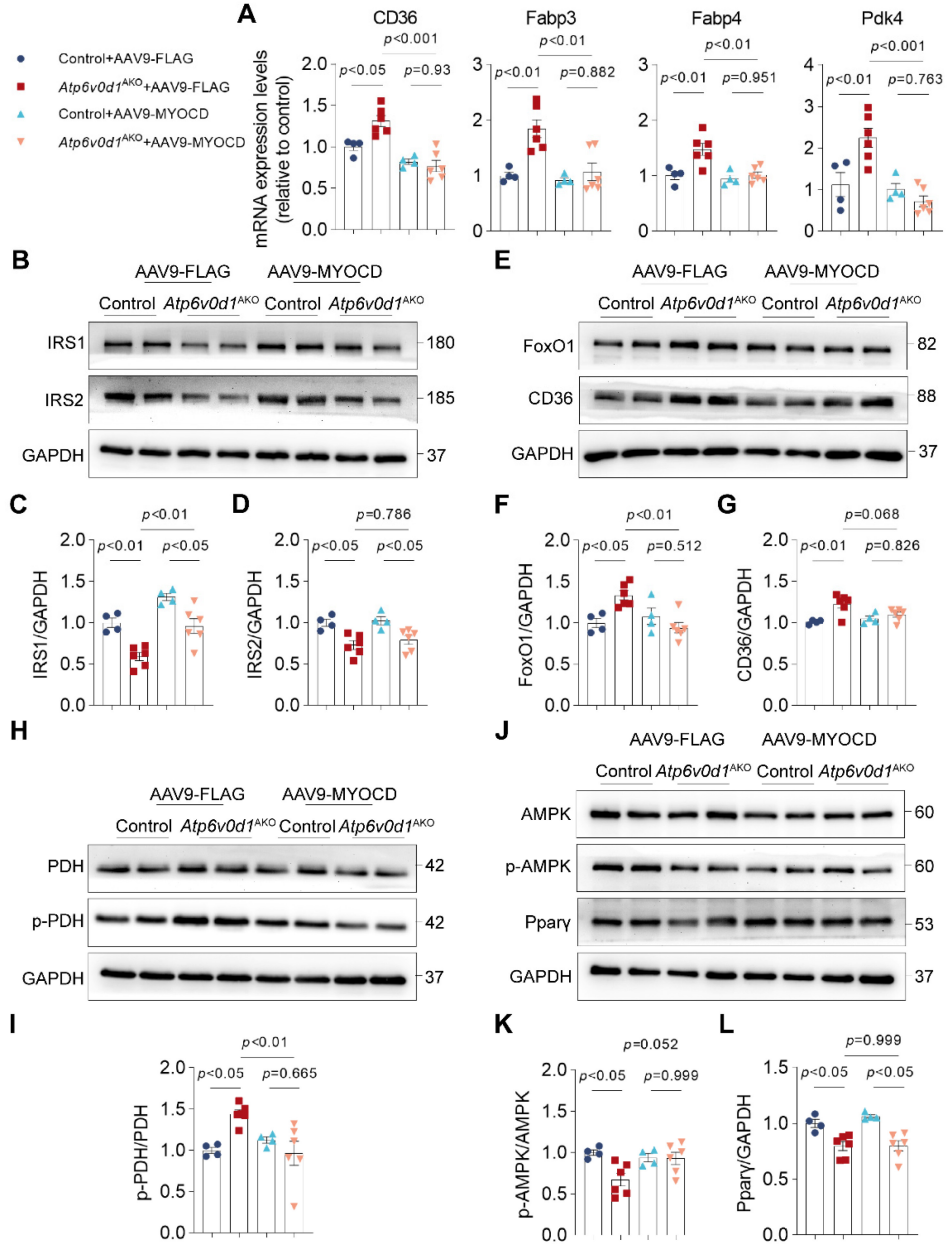
** Increased myocardin expression ameliorates metabolic reprogramming in *Atp6v0d1*^AKO^ mice. A**, qPCR quantification of the mRNA levels of FAO-related genes from hearts of control and *Atp6v0d1*^AKO^ hearts after tail vein injection of AAV9-cTnt-myocardin or AAV9-vectors. **B-D**, Representative immunoblots and quantification of IRS1 and IRS2 protein levels from heart tissues of control and *Atp6v0d1*^AKO^ mice after tail vein injection of AAV9-cTnt-myocardin or AAV9-vectors. Representative immunoblots of two independent experiments are shown. **E**, Representative immunoblots of FoxO1, and CD36 protein levels from heart tissues of control and *Atp6v0d1*^AKO^ mice after tail vein injection of AAV9-cTnt-myocardin or AAV9-vectors. Representative immunoblots of two independent experiments are shown. **F-G**, Quantification of specified densitometric ratios. **H**, Representative immunoblots of p-PDH and PDH protein levels from heart tissues of control and *Atp6v0d1*^AKO^ mice after tail vein injection of AAV9-cTnt-myocardin or AAV9-vectors. Representative immunoblots of two independent experiments are shown. **I**, Quantification of pPDH/PDH ratio. **J**, Representative immunoblots of p-AMPK, AMPK and Pparp-γ protein levels from heart tissues of control and *Atp6v0d1*^AKO^ mice after tail vein injection of AAV9-cTnt-myocardin or AAV9-vectors. Representative immunoblots of two independent experiments are shown. **K-L**, Quantification of specified densitometric ratios. Data represent the mean ± SEM (n = 4 - 6) (one-way ANOVA).

Given the critical role of disturbed cardiac metabolism in the development of cardiomyopathy, we decided to investigate the contribution of myocardin downregulation to metabolic disorders in *Atp6v0d1*^AKO^ mouse hearts. Indeed, we found that restoring myocardin expression normalized the expression of genes involved in metabolic reprograming in *Atp6v0d1*^AKO^ hearts, where the mRNA levels of *Pdk4*, *CD36*, *Fabp3* and *Fabp4* were significantly decreased (Figure 6A). Given the critical role of insulin signaling in regulating cardiac metabolism, we explored how myocardin regulates IRS expression in *Atp6v0d1*^AKO^ mouse hearts. The results showed that myocardin upregulation increased the expression of IRS-1 (Figure 6B-6D). However, the expression of IRS-2 was not restored by myocardin upregulation (Figure 6B-6D), and serum insulin levels suggested that myocardin upregulation may not alter systemic insulin resistance (Figure S8G). In addition, the protein levels of FoxO1, CD36, p-PDH and p-AMPK were restored in *Atp6v0d1*^AKO^ hearts (Figure 6E-6L). However, myocardin upregulation had no effect on PPARγ expression (Figure 6J and 6L). Previous studies demonstrated that PPARγ deficiency induced cardiac hypertrophy without interfering with cardiac contractile function 36. Although myocardin upregulation completely restored cardiac contractile function in *Atp6v0d1*^AKO^ mice (Figure 5H and 5I), the cardiac hypertrophy was ameliorated but not completely corrected, which may be attributed to the effect of PPARγ that was not counteracted by myocardin upregulation.

### Myocardin is critical for transcriptional activation of IRS-1

**Figure 7:**
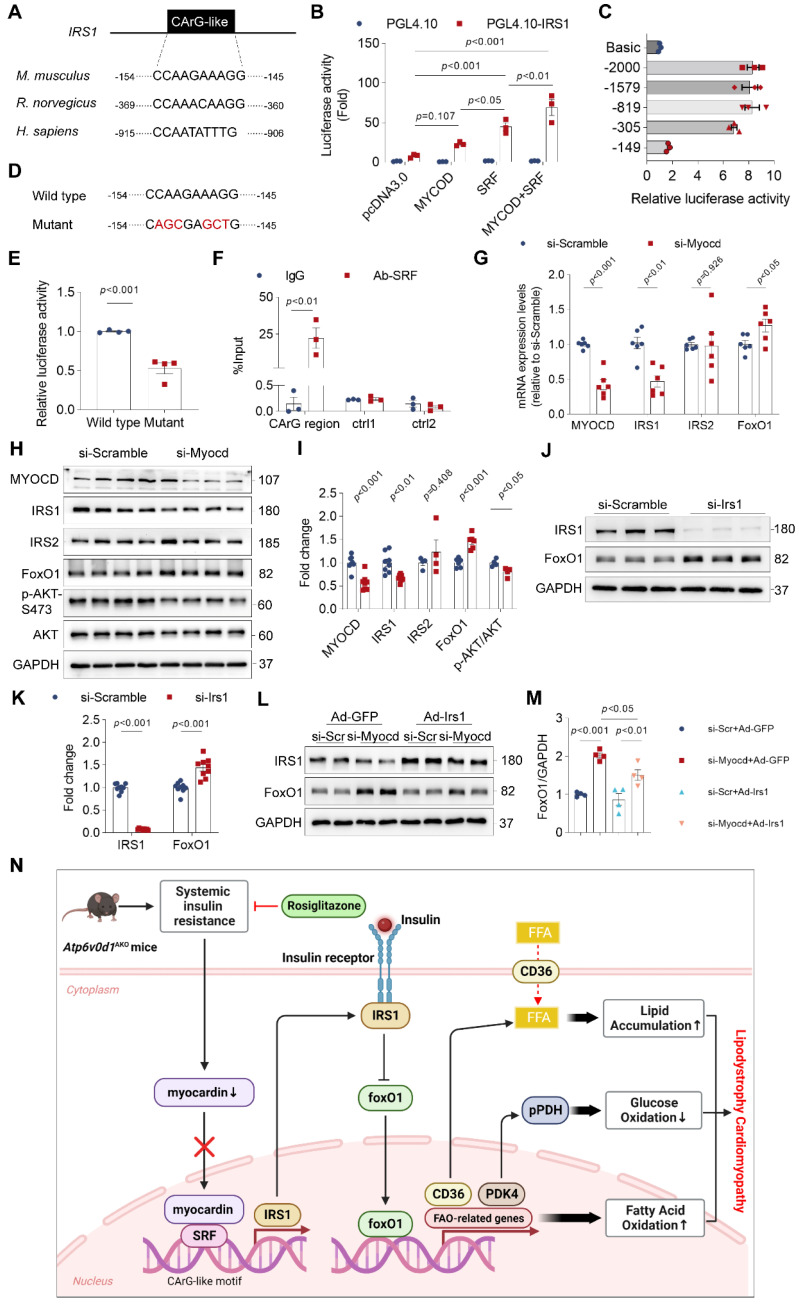
** Myocardin is critical for transcriptional activation of IRS-1. A**, The IRS1 promoter contains the conserved CArG-like element. **B**, Firefly luciferase activities detected in HEK293T cells transfected with promoterless pGL4.10 or vectors expressing IRS1 promoter in the presence of SRF or pcDNA3.0 and using Renillin as a normalization control. Results are depicted as fold of change in activity compared to pGL4.10 alone. Data represent the mean ± SEM (n = 3) (one-way ANOVA). **C**, The promoter activities of rat IRS1 promoter truncations were determined via luciferase assay. **D**, Mutation of the conserved CArG-like element in IRS1 promoter. **E**, Firefly luciferase activities of wild type and mutated IRS1 promoter. Data represent the mean ± SEM (n = 3) (two-tailed unpaired Student's *t*-test). **F**, IgG and SRF ChIP-PCR of rat neonatal cardiomyocytes. Enrichment of the IRS1 promoter following pull-down is normalized to input. Data represent the mean ± SEM (n = 3) (one-way ANOVA). **G**, qPCR quantification of the mRNA levels of myocardin, IRS1, IRS2 and FoxO1 in rat neonatal cardiomyocytes infected with myocardin siRNA or scrambled siRNA. Data represent the mean ± SEM (n = 6) (two-tailed unpaired Student's *t*-test). **H**, Representative immunoblots of myocardin, IRS1, IRS2 and FoxO1 protein levels in rat neonatal cardiomyocytes infected with myocardin siRNA (si-Myocd) or scrambled siRNA (si-Scramble) as a control. **I**, Quantification analysis of indicated protein levels. Data represent the mean ± SEM (n = 4 - 8) (two-tailed unpaired Student's *t*-test). **J**, Representative immunoblots of IRS1 and FoxO1 protein levels in rat neonatal cardiomyocytes infected with IRS1 siRNA (si-Irs1) or scrambled siRNA (si-Scramble) as a control. **K**, Quantification analysis of IRS1 and FoxO1 protein levels. Data represent the mean ± SEM (n = 9) (two-tailed unpaired Student's *t*-test). **L,** Representative immunoblots of IRS1 and FoxO1 protein levels in rat neonatal cardiomyocytes infected with myocardin siRNA (si-Mycod) or scrambled siRNA (si-Scr) and Ad-IRS1 or Ad-GFP. **M**, Quantification analysis of FoxO1 protein levels. Data represent the mean ± SEM (n = 4) (one-way ANOVA).** N**, Schematic illustration of the working model for the reduced expression of myocardin in *Atp6v0d1*^AKO^ hearts resulting in the decline in IRS-1 expression and the development of lipodystrophy cardiomyopathy. We discovered that myocardin enhances the interaction between SRF and the CArG element, resulting in the transcriptional activation of IRS-1. *Atp6v0d1*^AKO^ hearts have reduced myocardin expression, leading to decreased IRS-1 expression and upregulation of the downstream factor FoxO1. The translocation of FoxO1 to the nucleus exacerbates fatty acid uptake and oxidation through the upregulation of CD36 and FAO-associated genes, while suppressing glucose oxidation by upregulation of PDK4, and subsequently phosphorylation of PDH. Treatment with Rosiglitazone or upregulated myocardin expression increased IRS-1 expression and restored the balance of genes involved in lipid and glucose metabolism. Hence, *Atp6v0d1* deletion in hearts establishes a novel murine model of lipodystrophy cardiomyopathy, offering a new approach to understanding the pathogenesis of clinical adipose-related cardiomyopathy.

Myocardin regulates the transcription of genes containing the CArG element. We conducted a thorough analysis of the IRS1 promoter utilizing the JASPAR database (http://jaspar.genereg.net/). The DNA analysis uncovered the existence of a CArG-like element within the promoter regions of IRS-1 in mice, rats, and humans (Figure 7A). This discovery implies an unexplored role of myocardin in regulating the insulin signal within the heart. To test this hypothesis, we cloned the promoter region of IRS-1 containing a CArG-like element into a luciferase reporter plasmid, and co-transfected HEK293T cells with a myocardin overexpression plasmid (Myocd-OE) alone or together with a SRF overexpression plasmid (SFR-OE). Cotransfection of the myocardin plasmid moderately increased the luciferase activities compared with that associated with the empty plasmid, whereas cotransfection with myocardin and SRF plasmids dramatically increased the luciferase activities (Figure 7B). Both deletion and mutation of the CArG-like element in the promoter region of IRS-1strongly suppressed the luciferase activities in HEK293T cells (Figure 7C-7E), confirming that the CArG-like element was a key cis-element for IRS1 transcription. Chromatin immunoprecipitation (ChIP)-qPCR assays further confirmed that SRF bound specifically to the CArG-like element in the IRS-1 promoter in neonatal rat ventricular myocytes (NRVMs) (Figure 7F). Moreover, we knocked down myocardin expression with siRNA targeting myocardin (si-Myocd) to investigate its ability to decrease IRS-1 expression in cultured NRVMs. As anticipated, myocardin knockdown decreased IRS-1 expression (Figure 7G-7I). Consistent with the notion that insulin resistance inhibited Akt activity, Akt phosphorylation was decreased in myocardin knockdown cells (Figure 7H and 7I). Collectively, these results indicated that myocardin directly regulates IRS-1 expression.

FoxO1 is a critical regulator of lipid and glucose metabolism. In myocardin knockdown cardiomyocytes, we also observed an increase in protein abundance of FoxO1 (Figure 7H and 7I). The results are consistent with our findings *in vivo* that increasing myocardin expression in hearts of *Atp6v0d1*^AKO^ mice decreased FoxO1 expression (Figure 6E and 6F). Since myocardin promotes gene transcription, the changes in FoxO1 expression upon manipulation of myocardin are not the direct effect of myocardin. Previous studies suggested that the activation of insulin signal inhibits cardiac FoxO1 expression/activity 37. To test the causal relationship between decrease in IRS-1 and increase in FoxO1 expression following myocardin knockdown, we examined the direct effect of IRS-1 knockdown on FoxO1 expression, and increasing IRS-1 on myocardin downregulation-induced FoxO1 increase. The data showed that FoxO1 protein abundance was increased following IRS-1 knockdown (Figure 7J and 7K). Adenovirus-mediated overexpression of IRS-1 in cardiomyocytes significantly suppressed the increased expression of FoxO1 induced by myocardin-knockdown (Figure 7L and 7M). The data collectively indicate IRS-1 mediates FoxO1 increase under the condition of myocardin downregulation.

## Discussion

In the current study, we identified Atp6v0d1 as a crucial regulator of adipogenesis, and showed that loss of adipose *Atp6v0d1* in mice resulted in a gradual loss of adipose tissues accompanied by systemic insulin resistance, hypercholesterolemia and hepatosteatosis, which are key features of generalized lipodystrophy 38, 39. However, the link between V_o_d1 and lipodystrophy in human has yet to be confirmed. Considering that deletion of any of the V-ATPase subunits all cause embryonic lethality, genetic mutations that severely affect V_o_d1 functions may not be discovered. In addition, we found that V_o_d1 expression was increased in the AT of HFD-fed mice (Figure S1H and S1I). Collectively, our data suggest V_o_d1 subunit plays an important role in regulating adipogenesis and AT functions. Here, we found that *Atp6v0d1*^AKO^ mice display a similar developmental trajectory of cardiomyopathy to that observed in *Bscl2*^-/-^ mice, the most widely used animal model for studying lipodystrophy-related cardiomyopathy recapitulating human type 2 Berardinelli-Seip congenital lipodystrophy (BSCL2) disease, which progresses from compensated cardiac hypertrophy to heart failure 40. Yet, loss of BSCL2 specifically in the myocardium also leads to the onset of cardiomyopathy 41, adding the complexity of this model as whether dysfunctional AT or altered cardiac functions as the direct cause for cardiomyopathy. Moreover, the cardiomyopathy in *Atp6v0d1*^AKO^ mice can be rescued by increasing systemic insulin sensitivity with Rosiglitazone (Figure S6), similar to that observed in *Bscl2*^-/-^ mice 42, demonstrating the prominent feature of lipodystrophy cardiomyopathy that had been observed in the well-established *Bscl2*^-/-^ lipodystrophy mice.

However, *Atp6v0d1*^AKO^ mice exhibit some distinct pathological changes in hearts compared with those in *Bscl2*^-/-^ mice. First, lipid deposition in the heart, a pathological change that is widely observed in patients with lipodystrophy cardiomyopathy, is evident in *Atp6v0d1*^AKO^ mice, whereas *Bscl2*^-/-^ cardiomyopathy is characterized by reduced lipid accumulation 40. Second, the heart energy expenditure in *Atp6v0d1*^AKO^ mice is shifted preferentially to fatty acid oxidation. This, together with lipid accumulation implicates the occurrence of “lipotoxic” cardiomyopathy in *Atp6v0d1*^AKO^ mice, which is in contrast to *Bscl2*^-/-^ cardiomyopathy mainly caused by hyperglycemia and glucotoxicity 43. Third, the cardiac hypertrophy in *Atp6v0d1*^AKO^ cardiomyopathy is not accompanied by activation of the fetal genes, *Nppa* and *Myh7*, even when the contractile function is impaired. There is evidence that lower levels of ANP are associated with increased muscle mass in overweight and obese patients compared with those with normal weight 44. Most obese patients with HF have comparatively low levels of natriuretic peptides 45, although the underlying mechanisms are unknown. From this perspective, *Atp6v0d1*^AKO^ cardiomyopathy demonstrated the distinctive characteristics of obese cardiomyopathy in humans. Previous studies revealed that myocardin strongly transactivates the promoter for ANP 46. Myocardin expression was decreased in *Atp6v0d1*^AKO^ cardiomyopathy, and mimicking myocardin downregulation in cardiomyocytes decreased ANP expression (Figure S9). Our findings suggest that decreased myocardin expression may explain the distinct HF phenotype in obesity cardiomyopathy. Taken together, these findings highlight the potential of the murine *Atp6v0d1*^AKO^ lipodystrophy model as a new approach to gain insights into the development and underlying mechanisms of adipose tissue-associated cardiomyopathy.

Insulin signaling in the heart plays a central role in regulating cardiac metabolism, maintaining normal cardiac functions and survival of cardiomyocytes 47. Regulation of IRS has major roles in the control of cardiac metabolic homeostasis and function 47. Loss of IRS-1 and IRS-2 in the heart following chronic insulin stimulation contributes to insulin resistance, abnormal lipid and glucose metabolism, and heart failure 48. Phosphorylation of IRS protein by p38α MAP kinase, JNK, mTOR, or protein kinase C (PKC) stimulates its degradation 49. However, transcriptional regulation of IRS, especially in myocardial tissues, remains to be elucidated. Here, we uncovered a previously unrecognized role of myocardin in regulating IRS-1 transcription. Through luciferase reporter and ChIP-qPCR assays, we revealed that myocardin facilitates the interaction of SRF with the conserved CArG element of the IRS-1 promoter region to promote transcriptional activation of IRS-1. Moreover, knockdown of myocardin expression decreased IRS-1 transcription. In the hearts of *Atp6v0d1*^AKO^ mice, IRS-1 and myocardin expression levels were concomitantly decreased, while restored myocardin expression increased IRS-1 protein abundance. These results highlighted the critical role of myocardin downregulation in promoting insulin resistance in *Atp6v0d1*^AKO^ cardiomyopathy.

In the hearts of *Atp6v0d1*^AKO^ mice, the expression levels of genes associated with FA uptake (*CD36*) and FA oxidation (*Acadl*, *Acadm*, *Acads*, *Cpt1b*, *Cpt1c*, *Acsl1*, *Fabp3* and *Fabp4*) were upregulated, resulting in a preferential shift of heart energy expenditure to FAO. Furthermore, PDK4 expression was increased and PDH was activated, suggesting that glucose oxidation is suppressed. Interestingly, myocardin upregulation largely restored the expression of the genes controlling FA uptake and oxidation, and glucose metabolism, suggesting a critical role for myocardin in causing lipid and glucose metabolism disorder in *Atp6v0d1*^AKO^ cardiomyopathy. Accumulating evidence indicates that FoxO1 limits glucose oxidation by upregulating *PDK4,* and increases FA uptake and oxidation through upregulation of *CD36* and FAO-associated genes 37.

FoxO1 expression was increased in *Atp6v0d1*^AKO^ heart tissue, and myocardin upregulation decreased FoxO1 expression to normal levels. Moreover, knockdown of myocardin expression in cardiomyocytes increased FoxO1 expression. Collectively, these data indicate that FoxO1 mediates myocardin downregulation-induced lipid and glucose metabolism disorders.

FoxO1 is a downstream target of insulin, whereby insulin inhibits cardiac FoxO1 expression/activity through the IRS-1/Akt signaling in order to prevent gluconeogenesis. Previous studies showed that FoxO1 expression/activity was increased in HFD-induced diabetic cardiomyopathy 50. We found FoxO1 upregulation in heart tissues of *Atp6v0d1*^AKO^ mice. However, a causal link between insulin resistance and increased FoxO1 expression following myocardin downregulation in *Atp6v0d1*^AKO^ mice remains to be established. Our data showed that knockdown of IRS-1 decreased FoxO1 expression. Moreover, increasing IRS-1 expression reversed myocardin downregulation-induced FoxO1 upregulation in cardiomyocytes. Therefore, the myocardin-mediated increase in FoxO1 expression is a result of myocardin-induced insulin resistance. Taken together, we propose that the myocardin inhibition-insulin resistance-increased FoxO1 expression signaling axis plays a central role in promoting lipid and glucose metabolism disorder and the development of cardiomyopathy in *Atp6v0d1*^AKO^ mice (Figure 7N).

In this study, we found that Rosiglitazone treatment restored myocardin expression in *Atp6v0d1*^AKO^ mice, suggesting that myocardin downregulation is a result of the systemic insulin resistance that occurs in diabetes, obesity and lipodystrophy. Indeed, we observed decreased myocardin expression in HFD-induced type 2 diabetic cardiomyopathy in mice. Even though myocardin downregulation may not be present in all types of metabolic cardiomyopathy, insulin resistance is prominent in the heart in these conditions 51. Thus, upregulating myocardin is implicated as a potential therapeutic strategy for metabolic cardiomyopathies. The therapeutic effect of myocardin on treatment of diabetic and obesity cardiomyocytes, as well as the mechanism underlying systemic insulin resistance and reduction of myocardin in heart warrants future study.

Interestingly, V-ATPase in cardiomyocytes has also been shown to participate in regulating cardiac biological process and metabolism. For example, the study by Liu and colleagues demonstrated that lipid oversupply impairs V-ATPase function in heart by promoting CD36-mediated lipid uptake, which feeds forward to enhanced CD36 translocation, leading to insulin resistance and contractile dysfunction 52. Activation of V-ATPase in the heart can ameliorate lipid-induced cardiomyopathy 53. More recently, a study by Li, et al. showed that V-ATPase subunit, ATP6AP2 has a critical role in regulating autophagic flux in heart 54. Reducing ATP6AP2 expression in cardiomyocytes impairs autophagic flux and subsequently activates NLRPs, resulting in cardiac dysfunction. At present, it remains unknown whether and how V_o_d1 subunit expressed in heart participate in regulation of cardiac function under physiological and pathological conditions. In a recent study, deletion of V_o_d2, an isoform of the V_o_d1 subunits in the heart indeed increased surface CD36 abundance in cardiomyocytes and increased cardiac fibrosis 53. Given the critical role of V_o_d1 subunit in adipogenesis, and deleting *Atp6v0d1* in adipocytes results in the pathogenesis of cardiomyopathy, it's intriguing to explore the effect of deleting *Atp6v0d1* in cardiomyocytes on cardiac function.

In summary, by deleting *Atp6v0d1* in adipocytes, we established a murine generalized lipodystrophy model that reproduces the major pathological features observed in patients with lipodystrophy and obesity cardiomyopathy. This model paves a new avenue for gaining insights into the pathogenesis of adipose dysfunction-related cardiomyopathy. Furthermore, we identified myocardin downregulation as a key mechanism of cardiac insulin resistance mediated by decreasing IRS-1 expression, highlighting the therapeutic potential of upregulating myocardin activity for lipodystrophy cardiomyopathy and insulin resistance-related HF.

## Supplementary Material

Supplementary figures and tables.
