# Supplementary material for: Myocardin reverses insulin resistance and ameliorates cardiomyopathy by increasing IRS-1 expression in a murine model of lipodystrophy caused by adipose deficiency of vacuolar H+-ATPase V0d1 subunit
Source: Theranostics. 2024 Mar 11;14(5):2246–64. doi: 10.7150/thno.93192 (PMC10945344; doi:10.7150/thno.93192)
Supplement: Supplementary file 1 — Supplementary figures and tables. [file thnov14p2246s1.pdf]

SUPPLEMENTARY MATERIAL

SUPPLEMENTARY FIGURE LEGENDS

Supplemental Figure 1. *Atp6V0d1* expression changes during differentiation in 3T3-L1 cells, and adipogenesis is affected by Atp6V0d1 inhibition. A-D, Fold change in expression of *Atp6V0d1*, *Pparg*, *Cebpa* and *Cebpb* genes during 3T3-L1 adipocyte differentiation. Gene expression is normalized by the geometric mean of the expression of *β-actin, Gapdh* and *36b4* (n = 3 per group). **E**, Representative western blot of ATP6V0D1 and PPAR-γ in 3T3-L1 adipocytes from Days 0–12 of differentiation (n = 3 per group). **F**, Representative image of lipid droplets stained by Oil Red O and fluorescent dye in 3T3-L1 adipocytes. **G**, Representative western blot of ATP6V0D1, PPAR-γ and CEBPα expression in 3T3-L1 adipocytes treated with shRNA targeting *Atp6v0d1* or control shRNA (n = 3 per group). **H-I**, Representative immunoblots and quantification of ATP6V0D1 protein levels in normal chow diet (NCD) and high fat diet (HFD) mice. A-D and I were analyzed by two-tailed unpaired Student's *t*-test. Data represent the mean ± SEM.

Supplemental Figure 2. Loss of adipose *Atp6v0d1* induces hepatosteatosis and lipodystrophy. **A**, Construction strategy for adipocyte-specific *Atp6v0d1* knockout mice (*Atp6v0d1*^{AKO}). **B**, Representative gel electrophoresis image showing genomic excision of *Atp6v0d1* region in adipose tissues of *Atp6v0d1*^{AKO}. **C**, Representative western blot of ATP6V0D1 in adipose tissues of control and *Atp6v0d1*^{AKO}. **D**, Representative images of control and *Atp6v0d1*^{AKO} mice. **E**, Representative images of liver, inguinal WAT (iWAT), epidermal white adipose tissue (eWAT), retroperitoneal WAT (rWAT), and brown adipose tissue (BAT). **F**, H&E staining of liver and adipose tissue sections. **G**, Plasma leptin. **H**, Plasma adiponectin. **I**, Liver weight. **J**, Hepatic cholesterol. **K**, Hepatic triglyceride. **L**, Hepatic non-esterified fatty acids. **M**, AST to ALT ratio.

Supplemental Figure 3. *Atp6v0d1*^{AKO} mice aged 36 weeks exhibit fatty liver,

lipodystrophy and insulin resistance. **A**, Plasma cholesterol. **B**, Plasma triglycerides. **C**, Plasma non-esterified fatty acids. **D**, Intraperitoneal glucose tolerance test (IPGTT). **E**, Insulin tolerance test (ITT). **F**, Liver weight.

Supplemental Figure 4. A, Heart rate data of control and *Atp6v0d1*^{AKO} mice in cardiac function examination by echocardiography in 8, 12, 16, 20, 24, 28 and 36 weeks. **B,** EF% and FS% of control and *Atp6v0d1*^{AKO} mice aged 8, 12, 16, 20, 24, 28 and 36 weeks. **C,** qPCR quantification of the mRNA levels of *Nppa*, *Nppb*, *Myh6* and *Myh7* from control and *Atp6v0d1*^{AKO} heart tissues. Data represent the mean ± SEM (n = 4 - 5 per group; two-tailed unpaired Student's t-test)

Supplemental Figure 5. A, Representative images of adult mice cardiomyocytes isolated from WT and *Atp6v0d1*^{AKO} hearts. **B,** Representative immunoblots of individual respiratory chain complexes.

Supplemental Figure 6. A, Differentially expressed genes analyzed by volcano plots between control and *Atp6v0d1*^{AKO} mice hearts. **B**, The top 10 (a ranking by P-value) significantly enriched GO terms in biological process group (BP), cellular component group (CC) and molecular function group (MF). **C**, The 15 most relevant Reactome pathways (https://reactome.org) according to the upregulated and downregulated subgroups of DEGs.

Supplemental Figure 7. Rosiglitazone ameliorates cardiomyopathy in *Atp6v0d1*^{AKO} mice. **A**, Schematic illustration of Rosiglitazone treatment (50 μg per 10 g mouse weight), delivered via intragastric gavage to control and *Atp6v0d1*^{AKO} mice. **B**, Echo measurement of left ventricular ejection fraction (EF%) and fractional shortening (FS%) of control and *Atp6v0d1*^{AKO} mice with or without Rosiglitazone treatment. **C**, Representative images of hearts from control and *Atp6v0d1*^{AKO} mice with or without Rosiglitazone treatment. **D-E**, Heart weight (HW)

to tibia ratio and lung weight to tibia ratio of control and *Atp6v0d1*^{AKO} mice with or without Rosiglitazone treatment. **F-H**, Representative immunoblots and quantification of IRS1 and IRS2 protein levels. Data are normalized to GAPDH. **I**, Representative immunoblots of FOXO1, AMPK, p-AMPK and PPARγ protein levels for each group. **J-L**, Quantification of specified densitometric ratios. M, Representative immunoblots of CD36, PDH and p-PDH protein levels. **N-O**, Quantification of specified densitometric ratios (n = 4–6 per group). Data represent the mean ± SEM (one-way ANOVA).

Supplemental Figure 8. A, Echocardiographic evaluation of systolic function of control and *Atp6v0d1*^{AKO} mice after tail vein injection of AAV9-cTnt-myocardin or AAV9-vectors. n = 4-6 mice per group. Data are presented as mean ± SEM. **B,** The myocardin protein expression in mice cardiomyocyte by immunostaining, cardiomyocyte size and fibrosis in *Atp6v0d1*^{AKO} mice after tail vein injection of AAV9-cTnt-myocardin or AAV9-vectors. Left: Immuno-fluorescence imaging of myocardin. Middle: Representative images of WGA stain. Right: Representative transverse sections with Masson trichrome staining. **C-D,** Representative immunoblots and quantification of IRS1 protein levels in NCD and HFD mice. Data represent the mean ± SEM (n = 4 per group; two-tailed unpaired Student's t-test). **E-F,** Representative immunoblots and quantification of IRS2 protein levels in NCD and HFD mice. Data represent the mean ± SEM (n = 3 per group; two-tailed unpaired Student's t-test).

Supplemental Figure 9. Representative immunoblots and quantification of ANP protein levels in rat neonatal cardiomyocytes infected with myocardin siRNA (si-myocardin) or scrambled siRNA (si-Scramble) as a control. Data represent the mean ± SEM (n = 7 per group; two-tailed unpaired Student's *t*-test)

Supplementary Table 1. Antibodies used in the study

Target	Species originated	Dilution	Brand	Catalog number
ATP6V0d1	Rabbit	1:2000	Abcam	ab202897
PPARγ	Rabbit	1:1000	CST	#2435
CEBPα	Rabbit	1:500	Proteintech	18311-1-AP
FASN	Rabbit	1:500	Proteintech	10624-2-AP
β-tubulin	Mouse	1:5000	Proteintech	66240-1-Ig
β-actin	Mouse	1:5000	Proteintech	60008-1-Ig
ANP	Rabbit	1:1000	Abclonal	A1609
αMHC	Rabbit	1:1000	ABclonal	A12964
βMHC	Rabbit	1:1000	Santa cruz	Sc-168678
CD36	Rabbit	1:1000	Abcam	ab133625
Glut1	Rabbit	1:1000	Proteintech	21829-1-AP
Glut4	Mouse	1:1000	Proteintech	66846-1-Ig
PDH	Rabbit	1:1000	Proteintech	18068-1-AP
p-PDH	Rabbit	1:1000	Absin	Abs140331
IRS1	Rabbit	1:1000	CST	#2390
IRS2	Rabbit	1:1000	CST	#3089
FoxO1	Rabbit	1:1000	CST	#2880
PPARα	Rabbit	1:1000	Abcam	ab215270
PPARδ	Rabbit	1:1000	Abcam	ab178866
Pgc1α	Rabbit	1:1000	Abcam	ab191838
AMPK	Rabbit	1:1000	CST	#5831
p-AMPK	Rabbit	1:1000	CST	#2535
PI3K	Rabbit	1:1000	CST	#4257
p-PI3K	Rabbit	1:1000	CST	#4228
AKT	Rabbit	1:1000	CST	#9272
p-AKT-S473	Rabbit	1:1000	CST	#9271

p-AKT-T308	Rabbit	1:5000	CST	#9275
MYOCD	Rabbit	1:1000	sigma-aldrich	SAB4200539
SRF	Rabbit	1:100 (ChIP)	CST	#5147
GAPDH	Rabbit	1:5000	CST	#2118

Supplementary Table 2. Primers used in the study

Gene		
Atp6v0d1-m	Forward	5’-CAATGCCATTCTGGTGGAC-3’
	Reverse	5’-TTTCGGATTATCTCGATGTTCA-3’
Pparg-m	Forward	5’-GAAAGACAACGGACAAATCACC-3’
	Reverse	5’-GGGGGTGATATGTTTGAACTTG-3’
Cebpa-m	Forward	5’-CAAGAACAGCAACGAGTACCG-3’
	Reverse	5’-GTCACTGGTCAACTCCAGCAC-3’
Cebpb-m	Forward	5’-CTGCGGGGTTGTTGATGT-3’
	Reverse	5’-ATGCTCGAAACGGAAAAGGT-3’
Fasn-m	Forward	5’-TGGGTTCTAGCCAGCAGAGT-3’
	Reverse	5’-ACCACCAGAGACCGTTATGC-3’
Scd1-m	Forward	5’-GCGATACACTCTGGTGCTCA-3’
	Reverse	5’-CCCAGGGAAACCAGGATATT-3’
Dgat1-m	Forward	5’-TTCCGCCTCTGGGCATT-3’
	Reverse	5’-AGAATCGGCCCACAATCCA-3’
Dgat2-m	Forward	5’-GGCGCTACTTCCGAGACTAC-3’
	Reverse	5’-TGGTCAGCAGGTTGTGTGTC-3’
Fabp4-m	Forward	5’-GGATGGAAAGTCGACCACAA-3’
	Reverse	5’-TGGAAGTCACGCCTTTCATA-3’
Fatp1-m	Forward	5’-AGGTCAATGAGGACACGATGGAG-3’
	Reverse	5’-CTGGTACATTGAGTTAGGGTCCAAC-3’
Lipe-m	Forward	5’-GCGCTGGAGGAGTGTTTTT-3’
	Reverse	5’-CGCTCTCCAGTTGAACCAAG-3’
Lpl-m	Forward	5’-GGGAGTTTGGCTCCAGAGTTT-3’
	Reverse	5’-TGTGTCTTCAGGGGTCCTTAG-3’
Plin1-m	Forward	5’-AACGTGGTAGACACTGTGGTACA-3’
	Reverse	5’-TCTCGGAATTCGCTCTCG-3’
Plin2-m	Forward	5’-GACCTTGTGTCCTCCGCTTAT-3’

	Reverse	5’-CAACCGCAATTTGTGGCTC-3’
Plin3-m	Forward	5’-GGAGGAACCTGTTGTGCAG-3’
	Reverse	5’-ACCATCCCATACGTGGAACT-3’
Adpoq-m	Forward	5’-GGAGAGAAAGGAGATGCAGGT-3’
	Reverse	5’-CTTTCCTGCCAGGGGTTC-3’
Pepck-m	Forward	5’-ATGTGTGGGCGATGACATT-3’
	Reverse	5’-AACCCGTTTTCTGGGTTGAT-3’
36b4-m	Forward	5’-ACTGGTCTAGGACCCGAGAAG-3’
	Reverse	5’-CTCCCACCTTGTCTCCAGTC-3’
β-actin-m	Forward	5’-CTAAGGCCAACCGTGAAAAG-3’
	Reverse	5’-ACCAGAGGCATACAGGGACA-3’
Gapdh-m	Forward	5’-AGCTTGTCATCAACGGGAAG-3’
	Reverse	5’-TTTGATGTTAGTGGGGTCTCG-3’
Myh6-m	Forward	5’-GCCCAGTACCTCCGAAAGTC-3’
	Reverse	5’-GCCTTAACATACTCCTCCTTGTC-3’
Myh7-m	Forward	5’-CGTGCCAATGACGACC-3’
	Reverse	5’-CACTGCCTCCTCTACTTCTG-3’
Nppa-m	Forward	5’-GCTTCCAGGCCATATTGGAG-3’
	Reverse	5’-GGGGGCATGACCTCATCTT-3’
Nppb-m	Forward	5’-GAGGTCACTCCTATCCTCTGG-3’
	Reverse	5’-GCCATTTCCTCCGACTTTTCTC-3’
Cd36-m	Forward	5’-ACAGACGCAGCCTCCT-3’
	Reverse	5’-GATTTCAGATCCGAACA-3’
Acadl-m	Forward	5’-TTTCCTCGGAGCATGACATTTT-3’
	Reverse	5’-GCCAGCTTTTTCCCAGACCT-3’
Acadm-m	Forward	5’-TGATGTGGCGGCCATTAAGA-3’
	Reverse	5’-GGGTTAGAACGTGCCAACAAGAA-3’
Acads-m	Forward	5’-AAGTTTGGATCCGCACAGCAG-3’
	Reverse	5’-CAAGCTTTGGTGCCGTTGAG-3’

Cpt1a-m	Forward	5’-CTCCGCCTGAGCCATGAAG-3’
	Reverse	5’-CACCAGTGATGATGCCATTCT-3’
Cpt1b-m	Forward	5’-GTCGCTTCTTCAAGGTCTGG-3’
	Reverse	5’-AAGAAAGCAGCACGTTCGAT-3’
Cpt1c-m	Forward	5’-GATTTTCTGTACGTCACCCCC-3’
	Reverse	5’-AGGTGGTCTTTTTCCACCCC-3’
Fasn-m	Forward	5’-GGAGGTGGTGATAGCCGGTAT-3’
	Reverse	5’-TGGGTAATCCATAGAGCCCAG-3’
Acsl1-m	Forward	5’-TGCCAGAGCTGATTGACATTC-3’
	Reverse	5’-GGCATACCAGAAGGTGGTGAG-3’
Acsl3-m	Forward	5’-AACCACGTATCTTCAACACCATC-3’
	Reverse	5’-AGTCCGGTTTGGAACTGACAG-3’
Acsl5-m	Forward	5’-TCCTGACGTTTGGAACGGC-3’
	Reverse	5’-CTCCCTCAATCCCCACAGAC-3’
Fabp3-m	Forward	5’-ACCTGGAAGCTAGTGGACAG-3’
	Reverse	5’-TGATGGTAGTAGGCTTGGTCAT-3’
Pk-m	Forward	5’-GTTCTCACGGAGTCTGGC-3’
	Reverse	5’-GAGTCACGGCAATGATAGG-3’
Pfk-m	Forward	5’-CTGTGGTCCGAGTTGGTAT-3’
	Reverse	5’-ACGTGGCCTCCCTGAT-3’
Hk1-m	Forward	5’-GGCAGATTGAGGAAACCC-3’
	Reverse	5’-GGATGCTCCGAACATAAGAA-3’
Hk2-m	Forward	5’-ATCGCCTGCTTATTCACG-3’
	Reverse	5’-CCATTGTCTGTCACCCTTACT-3’
Pdk1-m	Forward	5’-GATGGCTATGAGAACGC-3’
	Reverse	5’-GTATTGTCTGTCCTGGTGAT-3’
Pdk2-m	Forward	5’-CTCCATCCGAATGCTAATC-3’
	Reverse	5’-TGGGAAGGGACATAGACC-3’
Pdk3-m	Forward	5’-CTCAACTGCTCCTCGTCC-3’

	Reverse	5’-GGCAAGCCATAACCAAAT-3’
Pdk4-m	Forward	5’-TGTGGTCCCTACAATGGC-3’
	Reverse	5’-AATGTGGCTTGGGTTTCC-3’
Cs-m	Forward	5’-AATCAGGAGGTGCTTGTCT-3’
	Reverse	5’-AGGAATAGCGAGGGTCAG-3’
Slc2a1-m	Forward	5’-CGTCGTTGGCATCCTTA-3’
	Reverse	5’-CCTTCTTCTCCCGCATC-3’
Slc2a4-m	Forward	5’-GCCTGCCCGAAAGAGT-3’
	Reverse	5’-TGAAGACCGTATTGACCACA-3’
Myocd-m	Forward	5’-ACACTCCTGGGGTCTGAACA-3’
	Reverse	5’-GCGGTATTAAGCCTTGGTTAGC-3’
FoxO1-m	Forward	5’-TCAAGGATAAGGGCGACAGC-3’
	Reverse	5’-CCTCCCTCTGGATTGAGCATC-3’
Irs1-m	Forward	5’-CGATGGCTTCTCAGACGTG-3’
	Reverse	5’-CAGCCCGCTTGTTGATGTTG-3’
Irs2-m	Forward	5’-CTGCGTCCTCTCCCAAAGTG-3’
	Reverse	5’-GGGGTCATGGGCATGTAGC-3’
Myocd-r	Forward	5’-GATAGTAAGAACCGCCACAA-3’
	Reverse	5’-TGAAGCAGCCGAGCAT-3’
Irs1-r	Forward	5’-CCTGGAGTATTATGAGAACGAG-3’
	Reverse	5’-CCGCAATGGCAAAGTGT-3’
Irs2-r	Forward	5’-CCCCTGTGGGTCGGATTT-3’
	Reverse	5’-TGAGTGATGAGGCTGGGTATG-3’
FoxO1-r	Forward	5’-GGATAAGGGCGACAGCAACA-3’
	Reverse	5’-TCTTGCCTCCCTCTGGATTG-3’
ChIP CArG region-r	Forward	5’-TTTTGATGGCAAGACGACTT-3’
	Reverse	5’-TTCTGTTCCCTAAGACACCTAT-3’
ChIP Ctrl1-r	Forward	5’-TAGCACCAACAGGACCCA-3’
	Reverse	5’-GCATCGTGAAGAAGGCATA-3’

ChIP Ctrl2-r	Forward	5’-AGCAACCGCAAAGGAAA-3’
	Reverse	5’-AGCAACTACCACTGGGAGAC-3’

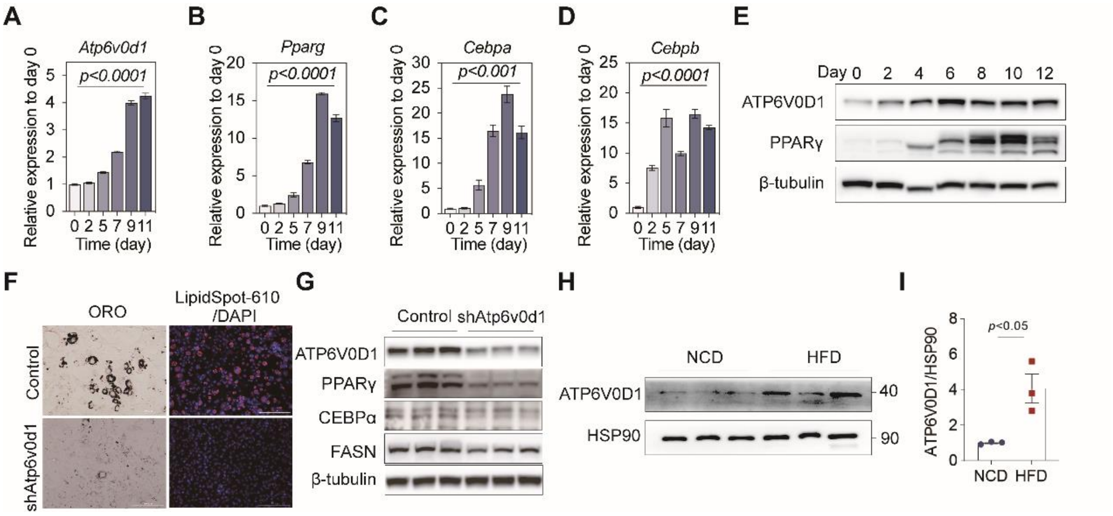

Supplementary Figure 1

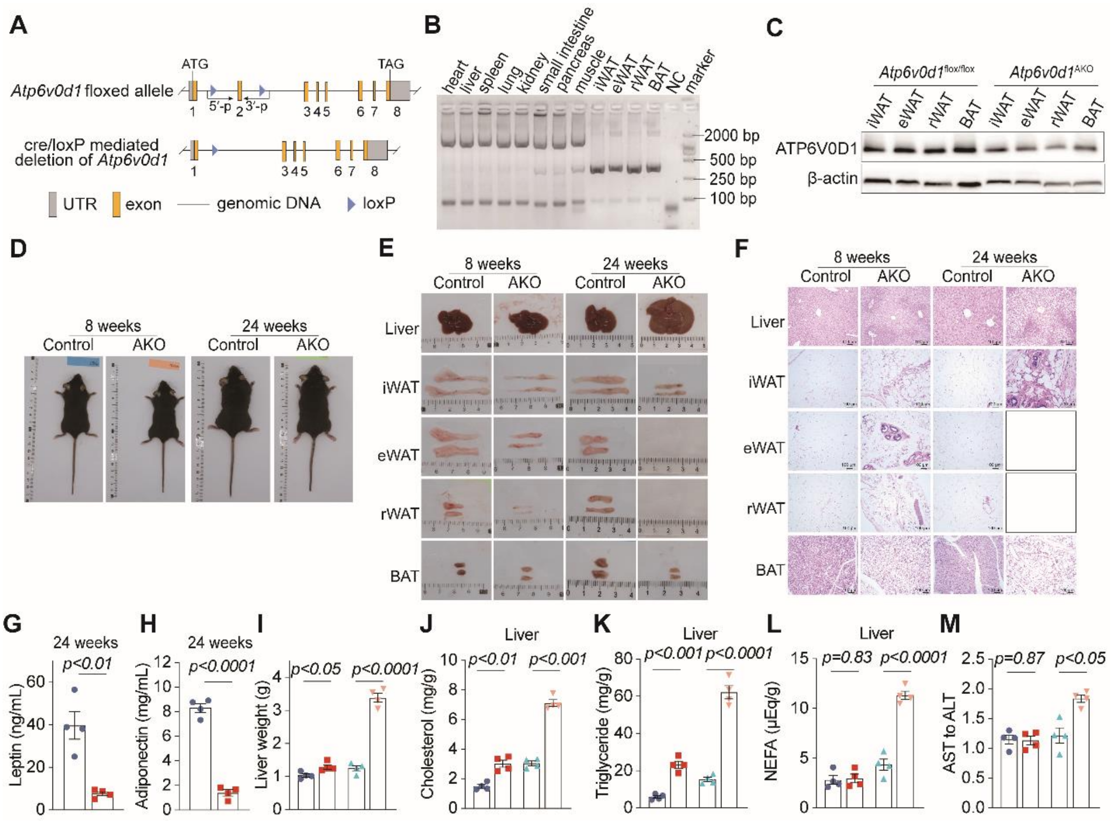

Supplementary Figure 2

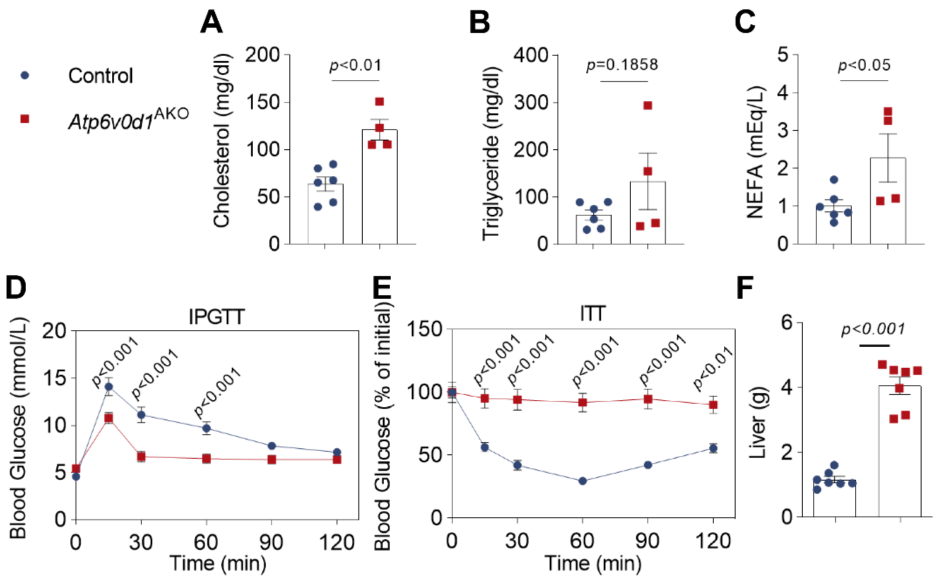

Supplementary Figure 3

A

Heart rate	Control	*Atp6v0d1*AKO
8 weeks	595.1±33.2	608.3±32.2
12 weeks	604.5±24.2	601.5±26.3
16 weeks	603±31.9	570±33.4
20 weeks	581.1±38.6	566.1±24.6
24 weeks	573.7±26.4	563.5±26.9
28 weeks	546.1±23.6	542.9±37.1
36 weeks	533.2±18.7	544.2±15.9

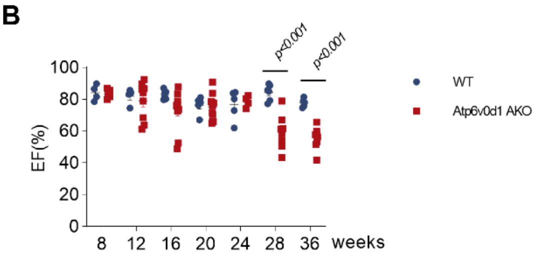

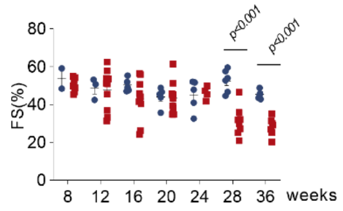

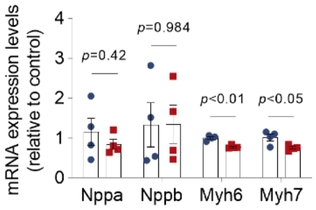

Supplementary Figure 4

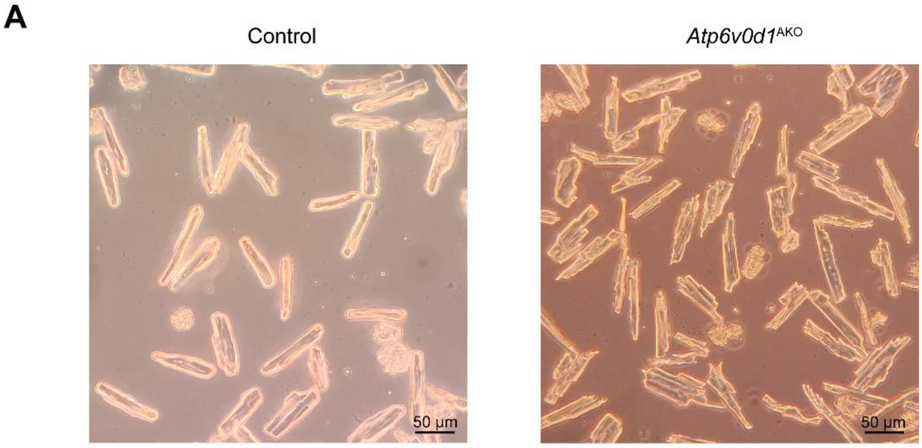

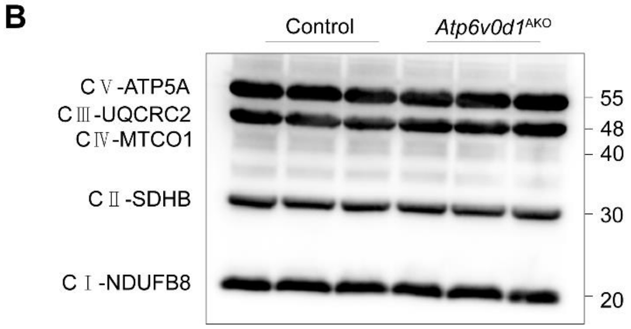

Supplementary Figure 5

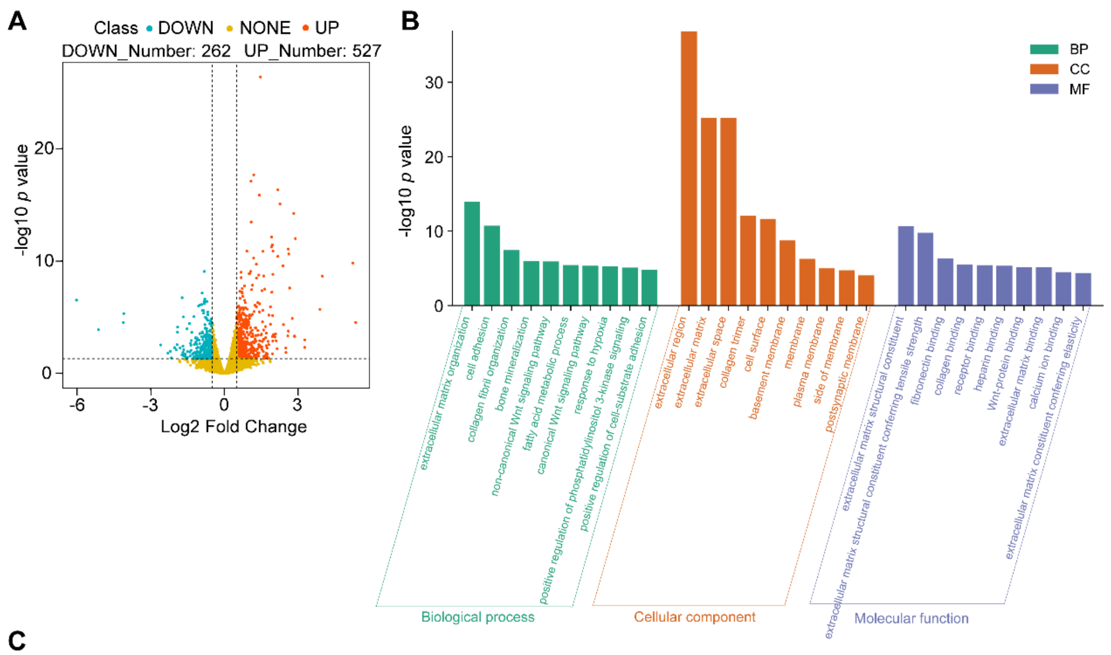

C

The 15 most relevant pathways sorted by *p* value using the upregulated and downregulated subgroups of DEGs.

Term	Input Number	Background Number	P-Value	Corrected P-Value
Upregulated Genes				
Extracellular matrix organization	39	263	2.81E-26	1.55E-23
Metabolism	71	1609	2.63E-17	7.27E-15
Degradation of the extracellular matrix	20	128	1.74E-14	3.22E-12
Collagen formation	16	78	1.85E-13	2.57E-11
Collagen degradation	14	55	4.78E-13	5.30E-11
Integrin cell surface interactions	15	70	6.09E-13	5.63E-11
Assembly of collagen fibrils and other multimeric structures	13	54	6.01E-12	4.76E-10
Collagen biosynthesis and modifying enzymes	13	57	1.08E-11	7.50E-10
Collagen chain trimerization	11	38	4.78E-11	2.94E-09
Metabolism of lipids	30	585	2.39E-09	1.32E-07
Fatty acid metabolism	16	167	5.14E-09	2.53E-07
Signal Transduction	68	2305	5.47E-09	2.53E-07
ECM proteoglycans	9	43	3.22E-08	1.37E-06
Hemostasis	25	497	7.38E-08	2.92E-06
Molecules associated with elastic fibres	8	35	1.05E-07	3.87E-06
Downregulated Genes				
Metabolism	27	1609	1.98E-05	6.75E-03
Attenuation phase	3	13	1.60E-04	2.72E-02
Receptor-type tyrosine-protein phosphatases	3	18	3.71E-04	3.27E-02
Neuronal System	9	314	3.85E-04	3.27E-02
HSF1-dependent transactivation	3	22	6.29E-04	4.28E-02
Interleukin-7 signaling	3	26	9.80E-04	4.85E-02
Regulation of HSF1-mediated heat shock response	4	61	9.99E-04	4.85E-02
IRS activation	2	6	1.24E-03	4.98E-02
Protein-protein interactions at synapses	4	66	1.32E-03	4.98E-02
Cytokine Signaling in Immune system	10	477	1.91E-03	6.06E-02
PI3K/AKT activation	2	9	2.40E-03	6.06E-02
Cellular response to heat stress	4	79	2.47E-03	6.06E-02
Cooperation of PDCL (PhLP1) and TRiC/CCT in G-protein beta folding	3	37	2.51E-03	6.06E-02
Protein folding	3	38	2.69E-03	6.06E-02
Chaperonin-mediated protein folding	3	38	2.69E-03	6.06E-02

Supplementary Figure 6

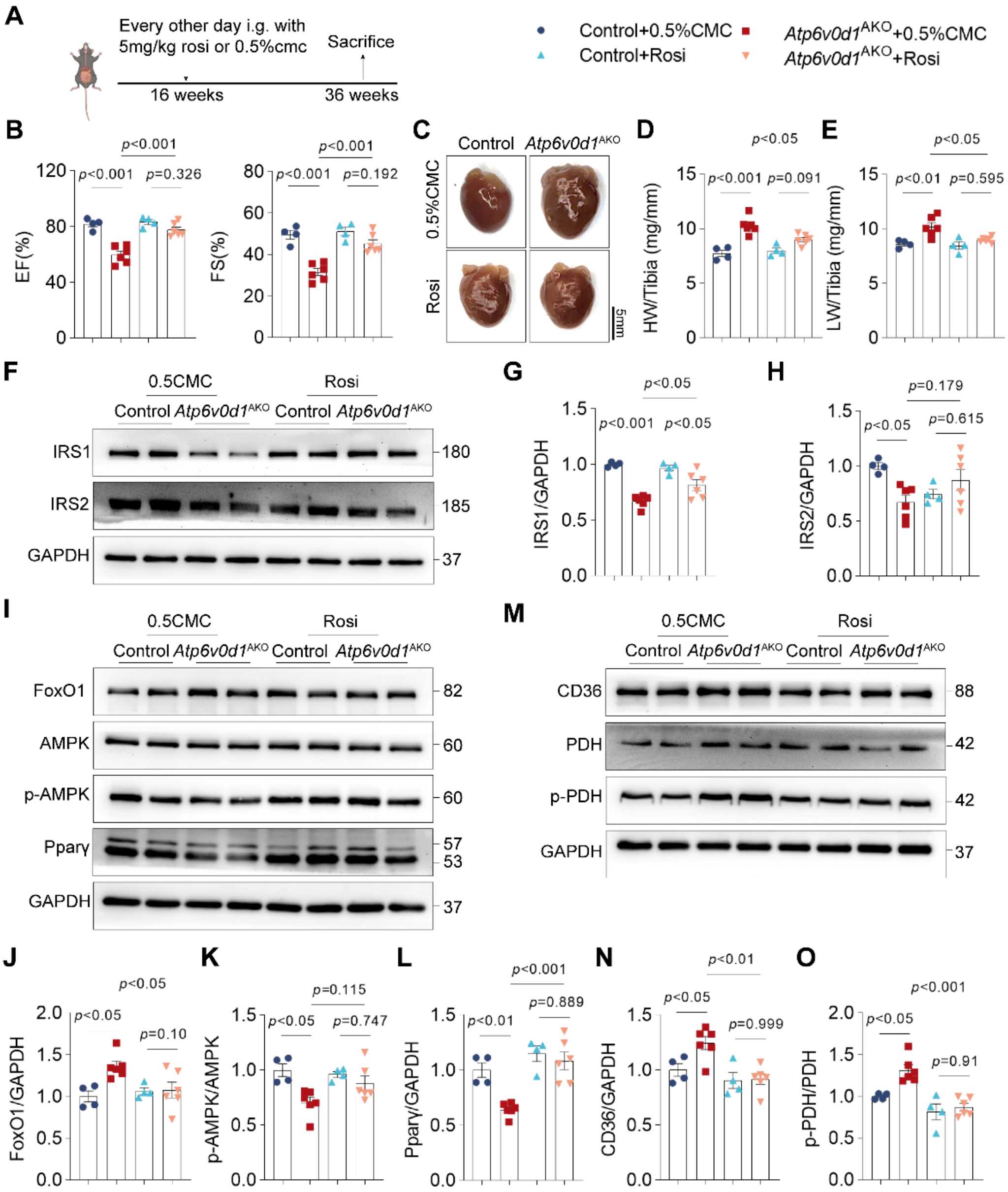

Supplementary Figure 7

A

	AAV9-FLAG		AAV9-MYOCD	
	Control	*Atp6v0d1*^AKO^	Control	*Atp6v0d1*^AKO^
LVAW;d	1.2±0.1	1.3±0.1	1.2±0.2	1.3±0.3
LVAW;s	1.9±0	1.8±0.2	1.9±0.1	1.8±0.3
LVID;d	3.3±0.2	4.1±0.5	3.4±0.5	3.4±0.3
LVID;s	1.6±0.2	2.8±0.4	1.8±0.4	1.8±0.2
LVPW;d	1±0.1	1±0.2	1±0.2	1±0.1
LVPW;s	1.6±0.1	1.4±0.2	1.7±0.1	1.7±0.1
EF	83±3	62.4±2.6	80.9±3.7	79.3±3.6
FS	50.8±3.3	33.3±1.8	48.6±3.5	47±3.6
LV Mass	139.7±16.3	205.3±28.6	144.8±7.5	160.1±16.8
LV Mass (Corrected)	111.8±13	164.2±22.9	115.9±6	128.1±13.4
LV Vol;d	43.7±7.3	77.1±21	50.3±17.6	48.1±9.8
LV Vol;s	7.5±2	29.2±8.7	10.1±5.1	10.1±3.3
Heart Rate	608.5±15.8	557.7±26.2	580.2±23.8	612.5±19.9

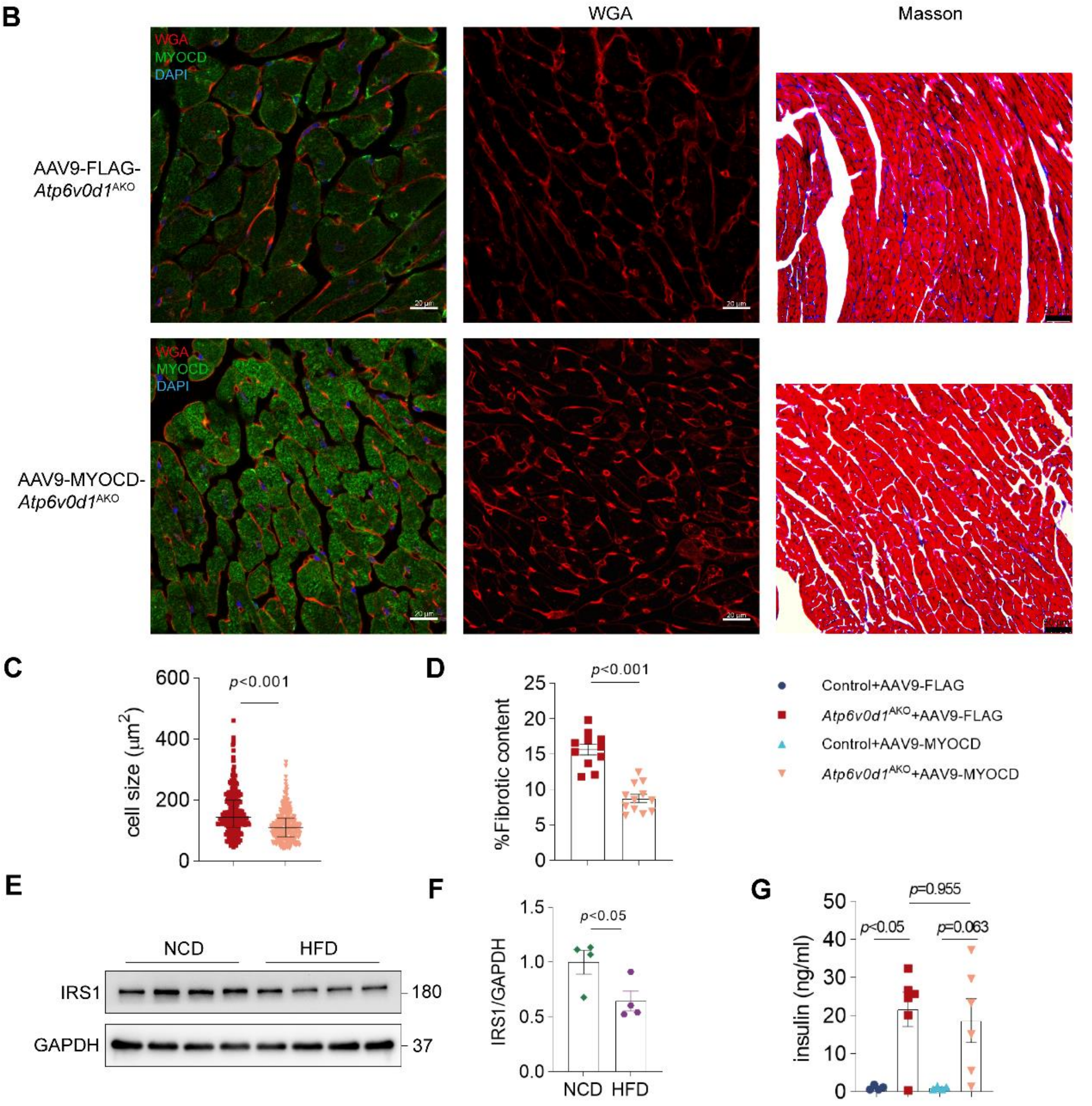

Supplementary Figure 8

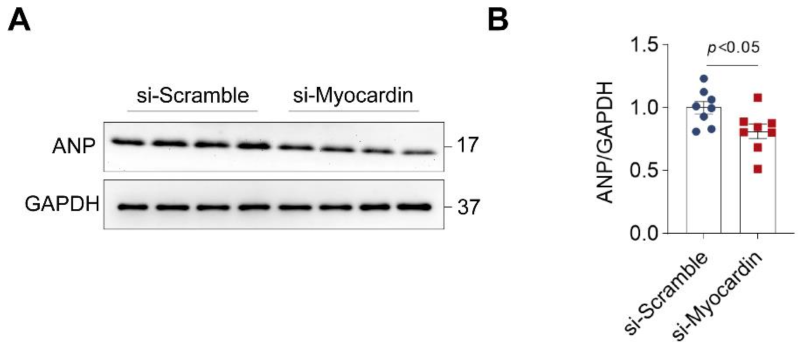

Supplementary Figure 9
